# Enhanced Antitumoral Activity and Photoacoustic Imaging Properties of AuNP‐Enriched Endothelial Colony Forming Cells on Melanoma

**DOI:** 10.1002/advs.202001175

**Published:** 2020-12-21

**Authors:** Paolo Armanetti, Anastasia Chillà, Francesca Margheri, Alessio Biagioni, Luca Menichetti, Giancarlo Margheri, Fulvio Ratto, Sonia Centi, Francesca Bianchini, Mirko Severi, Rita Traversi, Daniele Bani, Matteo Lulli, Tommaso Del Rosso, Alessandra Mocali, Elisabetta Rovida, Mario Del Rosso, Gabriella Fibbi, Anna Laurenzana

**Affiliations:** ^1^ Institute of Clinical Physiology (IFC) National Research Council Pisa 56124 Italy; ^2^ Department of Experimental and Clinical Biomedical Sciences University of Florence Florence 50134 Italy; ^3^ Institute for Complex Systems National Research Council Sesto Fiorentino 50019 Italy; ^4^ Institute of Applied Physics “N. Carrara” National Research Council Sesto Fiorentino 50019 Italy; ^5^ Department of Chemistry “Ugo Schiff” University of Florence Sesto Fiorentino 50019 Italy; ^6^ Department of Clinical and Experimental Medicine University of Florence Florence 50134 Italy; ^7^ Department of Physics Pontifícia Universidade Católica do Rio de Janeiro Rio de Janeiro 22451‐900 Brazil

**Keywords:** endothelial colony forming cells, gold nanoparticles, melanoma, nanomedicine, photoacoustic imaging

## Abstract

Near infrared (NIR)‐resonant gold nanoparticles (AuNPs) hold great promise in cancer diagnostics and treatment. However, translating the theranostic potential of AuNPs into clinical applications still remains a challenge due to the difficulty to improve the efficiency and specificity of tumor delivery in vivo as well as the clearance from liver and spleen to avoid off target toxicity. In this study, endothelial colony forming cells (ECFCs) are exploited as vehicles to deliver AuNPs to tumors. It is first demonstrated that ECFCs display a great capability to intake AuNPs without losing viability, and exert antitumor activity per se. Using a human melanoma xenograft mouse model, it is next demonstrated that AuNP‐loaded ECFCs retain their capacity to migrate to tumor sites in vivo 1 day after injection and stay in the tumor mass for more than 1 week. In addition, it is demonstrated that ECFC‐loaded AuNPs are efficiently cleared by the liver over time and do not elicit any sign of damage to healthy tissue.

## Graphical abstract

The findings herein firmly establish gold nanoparticle (AuNP)‐loaded endothelial colony forming cells (ECFCs) as excellent theranostic agents that can be tracked with simple imaging methods and display inherent antitumor properties. The study highlights their capacity to migrate to tumor sites in vivo 1 day after injection and remain in the tumor mass for more than 1 week.

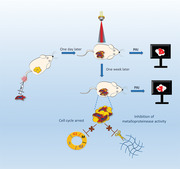

## 1 Introduction

With over 10 million new cases every year worldwide, metastatic cancer represents a difficult disease to treat and a major cause of morbidity and mortality.^[^ ^1^, ^2^ ^]^ Numerous studies have led to a better understanding of cancer at the genetic, molecular, and cellular levels, paving the way for new targets and strategies for treatment.^[^ ^3^, ^4^, ^5^ ^]^ Meanwhile, much focus has been made to develop new tools for cancer diagnosis.^[^ ^6^, ^7^ ^]^ Advances in cellular and molecular imaging have led to the development of various nanoparticle‐based diagnostic and/or imaging agents for the detection of cancer.^[^ ^6^, ^8^, ^9^, ^10^ ^]^ Nevertheless significant attempts are now being made to improve therapeutic and diagnostic properties in a single effective nanomedicine solution: this concept, coined as "theranostics," comprises selective nanosystems that are able to diagnose, deliver, and monitor the therapeutic response.^[^ ^11^, ^12^, ^13^, ^14^ ^]^

Gold nanoparticles (AuNPs), due to their innate bioinertness, quantum‐size‐related properties, such as highly tunable optical properties (e.g., plasmonically resonant optical absorbance and fluorescence or Raman scattering enhancement),^[^ ^15^ ^]^ have shown remarkable promise in cancer diagnostics and treatment. Indeed, as intrinsic theranostic agents, AuNPs stand out because of their remotely activatable anticancer activity (photothermal ablation)^[^ ^16^, ^17^ ^]^ and optical or hybrid imaging features (photoacoustic imaging, PAI),^[^ ^18^ ^]^ which removes the need of other nanocarriers to rely on additional therapeutic and diagnostic agents. AuNPs display optical absorption and scattering cross‐sections that are orders of magnitude greater than those of organic dyes, making them ideal contrast agents for a number of applications in biomedical optics, such as PAI.

However, despite the number of reports on the successful demonstration of Au nanomaterials for cancer theranostics^[^ ^19^, ^20^ ^]^ and their biomedical applications,^[^ ^21^ ^]^ their massive accumulation in the liver and spleen due to resident macrophages that form the mononuclear phagocyte system^[^ ^22^, ^23^ ^]^ is still an issue. Even though much effort is being focused on functionalizing theranostic nanoparticles with targeting moieties, several studies have shown that the presence of specific ligands does not always result into an increased accumulation in the tumor site, since it might increase the formation of a protein corona hiding the nanoparticle targeting ability.^[^ ^24^, ^25^, ^26^ ^]^ Based on these considerations, solutions able to boost the delivery of AuNPs to the target site are still much needed. Of particular interest is the observation that certain types of stem and immune cells, which have an innate ability to target and infiltrate tumors, can be utilized as vectors to deliver several types of anticancer payloads.^[^ ^27^, ^28^, ^29^, ^30^ ^]^ A contribution to these findings came from our recent study showing that endothelial colony forming cells (ECFCs)^[^ ^31^ ^]^ can be utilized to exploit the photothermal efficacy of near infrared (NIR)‐resonant chitosan‐coated AuNPs (ChAumix).^[^ ^32^ ^]^

In this study, we explored the antitumor effects and the tumor‐homing efficiency of AuNP–ECFCs following single intravenous injection into tumor‐bearing mice and we assessed their biodistribution in freshly excised mice organs at different time points post administration by exploiting the PAI properties of AuNP‐enriched ECFCs.

It is worth emphasizing that a crucial parameter that governs the effectiveness of cell‐based nanoparticle delivery is the payload capacity.^[^ ^33^ ^]^ Since rapid recycling and exocytosis of internalized nanoparticles can result in low loading capacity when relying on nonspecific endocytosis, we compared the payload capacity of ECFCs with that of more common models of tumor tropic cells, such as murine macrophages RAW264.7 and mesenchymal stem cells (MSCs), respectively. Nevertheless, we found that AuNPs are eagerly taken up by ECFCs without impairing their viability or cellular functions, and promoted unexpected antitumoral activities on melanoma cells.

We demonstrate that i) the loading capacity of AuNPs in ECFCs enhances the photoacoustic (PA) signal detection and allows a spectral tunability; ii) AuNPs happen to improve the unforeseen anticancer activity of ECFCs in vitro and in vivo; iii) ECFCs efficiently deliver AuNPs to tumors in mice; iv) ECFC‐loaded AuNPs are efficiently cleared by the liver over time, thus creating the premises for clinical translation.

## 2 Nanoparticle Uptake

### 2.1 Kinetics and Mechanism of Gold Nanoparticle Uptake

**Figure 1:**
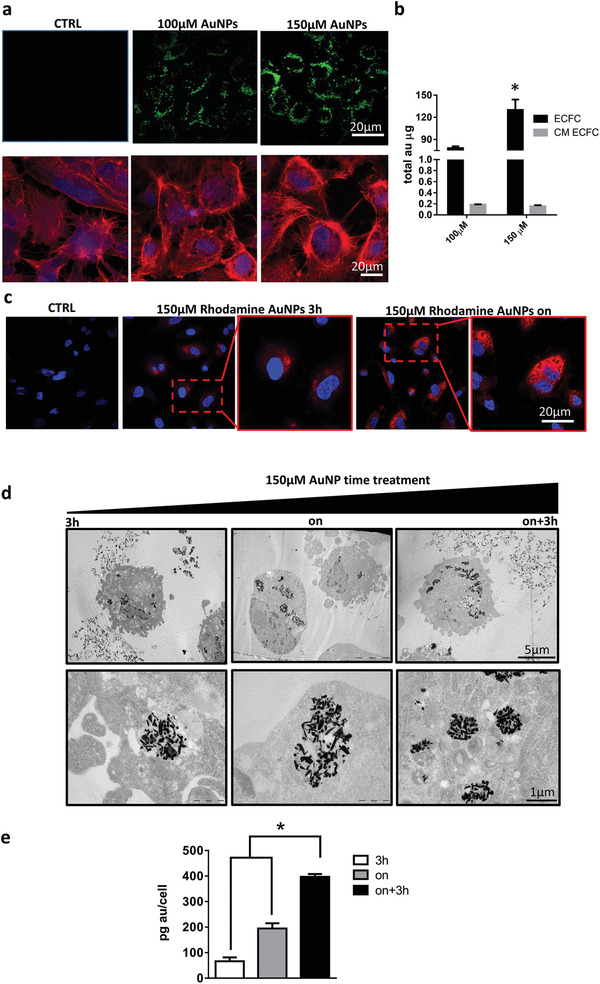
Uptake of chitosan‐capped Aumix by ECFCs. a) (Top row) Representative MPL images of ECFCs treated with increasing concentration of AuNPs; (bottom row) fluorescence microscopy of ECFCs labeled with phalloidin (RED), which stains cytoskeletal F‐Actin, and treated overnight with increasing dose of AuNPs (black dots). b) ICP‐AES analysis of gold content in 1.2 × 10^6^ ECFCs and derived culture media (CM) of cells treated overnight (on) with increasing dose of AuNPs. Statistical analysis was performed using unpaired Student's *t*‐test; error bars: mean ± SD (^*^ *p* < 0.05). c) Confocal microscopy of ECFCs treated for 3 h or overnight with AuNPs conjugated with rhodamine. d,e) Transmission electron micrographs and ICP‐AES analysis of ECFCs treated with one dose or two consecutive doses of AuNPs in 15 h. Significance was assessed by one‐way ANOVA test followed by Newman–Keuls post test. Error bars indicate mean ± SD; asterisk (^*^ *p* < 0.05) indicates significant differences between cells treated with a double or a single dose of AuNPs. All experiments were performed independently and at least in triplicate.

We previously demonstrated that ECFCs robustly internalize AuNPs without eliciting cell toxicity.^[^ ^32^ ^]^ Here, we monitored the nanoparticle location within the cells by using multiphoton luminescence (MPL) microscopy and confocal microscopy. Because gold nanoparticles exhibit MPL conversion, there is no need for secondary labeling. Representative MPL images of ECFCs treated with increasing concentration of AuNPs are shown in the upper row of **Figure** **1**a. The MPL emission from the metal nanoparticles is clearly visible as green dots around the nucleus, and shows a clear dose dependence. Results were confirmed by an analysis performed with more standard optical tools. In the lower row of Figure 1a and Figure S1 in the Supporting Information fluorescence microscopy of ECFCs labeled with cytoskeletal F‐Actin staining phalloidin revealed that AuNPs accumulated and formed intracellular aggregates (black spots) in perinuclear areas, which increased in density with dose, as previously described.^[^ ^32^ ^]^ The cellular uptake of AuNPs was quantitatively evaluated using inductively coupled plasma‐atomic emission spectroscopy (ICP‐AES) after incubating ECFCs with AuNPs at the concentrations of 100 × 10^−6^ and 150 × 10^−6^ m Au overnight (on) (Figure 1b). High amounts of AuNPs were internalized by the cells with a clear increase with dose. For both doses, only a negligible fraction of gold was found to remain in the culture media. Thus, the majority of AuNPs were taken up by the cells. AuNPs were then conjugated with rhodamine and confocal microscopy was used to assess the kinetics of uptake and retention within ECFCs. For uptake, ECFCs were incubated with AuNPs at a concentration of 150 × 10^−6^ m Au for 3 h and overnight (15 h). Figure 1c shows that the amount of AuNPs internalized by ECFCs is moderate after 3 h and significantly increases after 15 h. In previous studies, cells were only subjected to a single treatment of nanoparticles with increasing concentrations. With the aim to prove that ECFCs are capable to internalize massive amounts of AuNPs, we investigated whether multiple treatments of ECFCs with AuNPs increase the intracellular load. In Figure 1d,e, transmission electron microscope (TEM) and ICP‐AES analyses confirmed that a double dose of AuNPs increased the intracellular load by almost twofold compared to ECFCs treated for 15 h and more than eightfold compared to 3 h.

### 2.2 Comparative Study of AuNP Uptake

**Figure 2:**
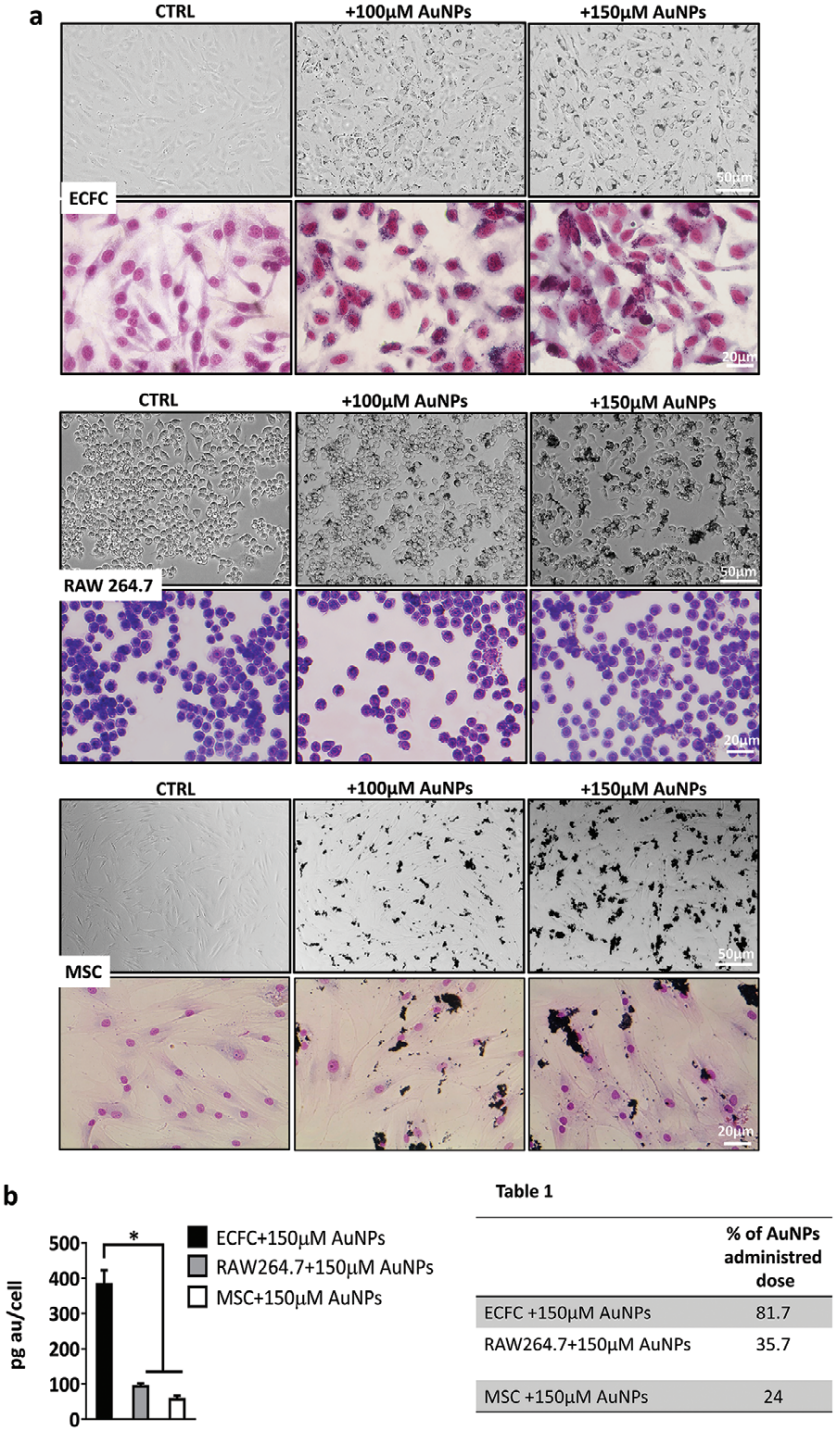
Comparative AuNP uptake in three different tumor tropic cell lines. a) Optical microscopy images of nonstained (upper panels) or May–Grunwald–Giemsa stained (lower panels) untreated (CTRL) or cells treated with increasing concentration of AuNPs. The inner gold content appears as black spots in the cells. Experiments were performed in triplicate. b) Percentage of administrated dose of AuNPs quantified by ICP.

Since MSCs and macrophages showed tropism to tumors^[^ ^27^, ^28^, ^29^, ^30^ ^]^ and thus, have been used as vehicles for cancer delivery, we investigated and quantified the amount of internalized AuNPs in these two cell lines using optical microscopy (**Figure** **2**a) and ICP‐AES (Figure 2b) and compared the results to those observed in ECFCs. Images in Figure 2a reveal that RAW264.7 and MSCs internalized much lower amounts of AuNPs than the ECFCs after overnight treatment. In particular, in the culture media of MSCs, we found massive aggregates of AuNPs that became more evident after May–Grunwald staining (lower row of Figure 2a). The results of the ICP‐AES analysis reported in Figure 2b, as pg of gold per cell and as percent of particles taken up by cells with respect to incubation dose (Table 1 in Figure 2), confirmed the microscopic observations. To summarize the results of both analyses, RAWs internalized particles better than MSCs, but ECFCs outclassed both models by as much as a factor of two and almost four, respectively. In quantitative terms, RAWs and MSCs were found to contain 36% and 24% of total available gold, respectively, while ECFCs incorporated up to 81% of AuNPs.

## 3 Gene Regulation in ECFCs Treated with AuNPs and ECFC/A375‐M6 Coculture

**Figure 3:**
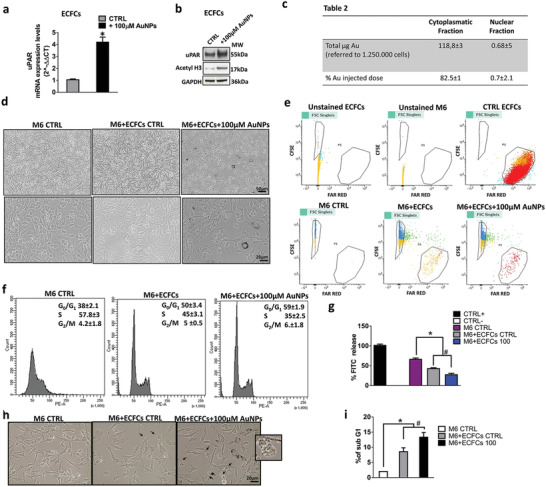
Assessment of antitumor activity of ECFCs on melanoma cells. a) mRNA levels of uPAR on untreated (CTRL) or AuNP‐treated cells as determined by qRT‐PCR analysis. The Student's *t*‐test was used to analyze the data. Error bars indicate mean ± SD; *n* = 3 experiments; ^*^ *p* < 0.05 indicates statistical significance. b) Western blot analysis of uPAR and acetylate H3. GAPDH was used as loading control. c) ICP‐AES analysis of nuclear and cytoplasmatic fraction of ECFCs treated with 100 × 10^−6^ m AuNPs. d) Optical microscopy images of melanoma (1 × 10^6^ CFSE‐A375‐M6 cells)/ECFC (5 × 10^4^ FAR RED‐ECFCs) coculture. e) Representative dot plots depicting FAR RED and CFSE fluorescence showing unstained and CFSE‐stained melanoma cells and unstained and FAR RED‐labeled ECFCS. f) Flow cytometry analysis of melanoma cells stained with propidium iodide. The percentage of cells in the different phases of the cell cycle was calculated by the ModFit program. g) Histograms of the collagenolytic activity of melanoma cells and melanoma ECFC coculture expressed as percentage of collagen degradation with respect to positive controls obtained by the addition of exogenous collagenase. Ctrl−: collagenolytic activity in the absence of cells and exogenous collagen; Ctrl+: collagenolytic activity in the absence of cells but in the presence of exogenous collagenase; M6 CTRL: collagenolytic activity in the presence of M6 cells; M6+ECFC, M6+ECFCs100 collagenolytic activity in the presence of coculture of M6+untreated ECFCs or +ECFC treated with 100 × 10^−6^ m AuNPs. h) Optical microscopy images of M6 (1 × 10^6^ CFSE‐A375‐M6 cells)/ECFC (1 × 10^5^ FAR RED‐ECFCs) coculture. Black arrows indicate the presence of apoptotic cells. i) Histograms representing the percentage of melanoma cells in sub‐G1 phase analyzed by FACS. Significance was assessed by one‐way ANOVA test followed by Newman–Keuls post test. Error bars indicate mean ± SD; asterisks (^*^ *p* < 0.05) indicate significant difference from the M6 untreated cells (CTRL). Hash signs (^#^ *p* < 0.05) indicate significant difference of M6 treated with AuNP–ECFC from M6 treated with ECFC CTRL. All experiments were performed independently and at least in triplicate.

We previously demonstrated the ability of AuNPs to modulate the expression of genes involved in cell motility, such as CXCR4. Indeed, unexpectedly, we found a significant upregulation of CXCR4 expression, and a sensible increase of cell migration indicating an enhanced tumor tropism of Au enriched cells.^[^ ^32^ ^]^ Interestingly, there was no obvious impact of AuNP treatment on cell viability,^[^ ^32^ ^]^ or cell morphology (Figure 2a). Here, we demonstrate that AuNPs are able to induce, at mRNA (**Figure** **3**a) and protein levels (Figure 3b), the expression of the urokinase plasminogen activator receptor (uPAR), which is known to play a critical role in the cell migration process. In light of these findings, we investigated whether the modulation of gene transcription related to chromatin modifications. Surprisingly, we found an increase of H3 acetylation in AuNP‐treated ECFCs. ICP‐AES analysis performed on nucleic and cytosolic fractions detected the presence of gold in the nuclei, which was calculated as 0.7% of incubation dose (Figure 3c). Next, in order to assess the translational value of ECFCs as vehicles of AuNPs for theranostic purposes, and to exclude the possibility that AuNP‐induced gene activation on ECFCs might support tumor growth and spreading, we investigated the effect of ECFC–AuNPs on melanoma cells through a coculture model (Figure 3d). For this purpose, 1 × 10^6^ carboxyfluorescein diacetate succinimidyl ester dye (CFSE)‐labeled A375‐M6 (M6) cells were treated with 5 × 10^4^ Far Red‐labeled ECFCs unloaded or loaded with AuNPs. The M6‐AuNP‐enriched ECFC coculture images reported in Figure 3e clearly show the ratio between the two populations. 24 h later, both cell cocultures were directed to a cell sorter analysis for CFSE and Far Red (Figure 3e). The two positive cell populations isolated from the two cocultures were clearly distinguished (Figure 3e). Sorted CFSE‐labeled melanoma cells were then stained with propidium iodide (PI) for cell‐cycle distribution analysis (Figure 3f). Untreated CFSE‐labeled melanoma cells (M6 CTRL) exhibited a relatively normal pattern, with most cells in the S phase (≈58%), and a lower G_0_–G_1_ phase (38%) peak of the cell cycle (Figure 3f). The treatment of M6 with ECFCs alone caused G_0_–G_1_ cell cycle arrest (50% vs 38% in M6 CTRL), whereas AuNP‐doped ECFCs led to a further increase of M6 cell population at the G_0_–G_1_ phase from 50% to 59%. This indicates that pretreatment of ECFCs with AuNPs enhances the cell cycle arrest. Melanoma cells display an invasive phenotype that allows them to degrade and infiltrate the extracellular matrix (ECM) largely composed of a dense, crosslinked network of collagen type I. To assess the effect of ECFCs on melanoma invasiveness, we performed a collagen degradation assay. As shown in Figure 3g, the presence of AuNP‐unloaded or loaded ECFCs on melanoma cell culture significantly blocks the degradation of collagen elicited by melanoma cells.

Furthermore, the increase of ECFC/M6 ratio in the cell cocultures was able to trigger apoptosis by inducing bleb formation, as indicated by the black arrows in Figure 3h and in its enlarged inset. The apoptotic effect of AuNP‐unloaded or loaded ECFCs on M6 cells was confirmed by fluorescent‐activated cell sorter (FACS) analysis with an increase of the percentage of cells in the sub‐G1 phase indicative of cell death (Figure 3i).

## 4 Photoacoustic Imaging of AuNPs–ECFC

### 4.1 Photoacoustic Imaging of AuNPs–ECFC in Test Objects

**Figure 4:**
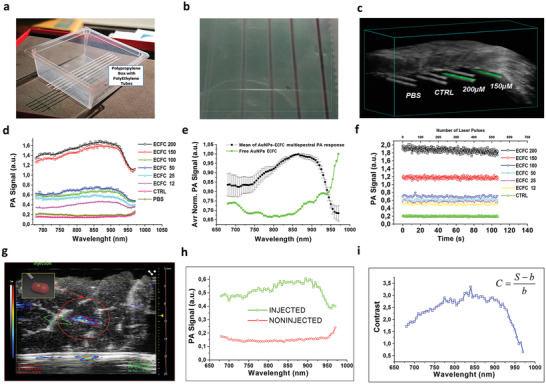
PA characterization of AuNP–ECFCs: a) photograph of the PA phantom. b) Polyethylene tubes filled with increasing concentration of AuNP–ECFCs up to 200 × 10^−6^ m and ECFC control, on the left end, c) 3D PA/US reconstruction of the PA phantom with a biological layer put above the polyethylene tubes; green scale: PA signal from the AuNP–ECFCs, grayscale: co‐registered US signal. d) Plot of the PA spectral trend of AuNP–ECFCs at different concentrations. e) Spectral fingerprint of the compound calculated by the normalization of PA signals from AuNP–ECFCs in comparison with those from blank ECFCs. f) Photostability of AuNP–ECFCs under laser illumination at fixed wavelength (860 nm) over time. g) Samples of chicken breast injected with a 50 µL bolus of AuNP–ECFCs; color scale: PA signal, grayscale: co‐registered US signal; h) PA spectral response of injected and noninjected regions; i) plot of contrast calculated as a function of wavelength.

Photoacoustic imaging was performed in vitro using polyethylene (PE) tubes (**Figure** **4**a) filled with increasing concentration of AuNP–ECFCs up to 200 × 10^−6^ m, ECFC control, and phosphate buffered saline (PBS) (Figure 4b,c). Typical PA patterns are reported in Figure 4, showing the multispectral responses of AuNP–ECFCs at different concentrations (12 × 10^−6^, 25 × 10^−6^, 50 × 10^−6^, 100 × 10^−6^, 150 × 10^−6^, and 200 × 10^−6^ m Au equivalent) (Figure 4d–f) and the signals provided from PBS (Figure 4c,d) and uncharged ECFCs (CTRL) dispersed in PBS (Figure 4c–f). The PA signal intensity shows a linear trend with AuNP concentration, with maximum intensity around 860 nm, in agreement with the plasmon absorption band position (Figure 4d,f). In order to identify relevant trends, the PA spectra acquired from all AuNP–ECFC samples were normalized and analyzed (Figure 4e) by calculating the percentage of variation (Figure S2, Supporting Information). We found variations in the range between 1% and 8%, with values under 4% for 70% of the data. This confirmed that the spectral trend was the same for all solutions (Figure 4e): the plasmon peak position and PA spectral shape from the loaded ECFC did not differ from reference solution of AuNP. The cellular clustering of the AuNPs did not modify their spectral trend (Figure S2b, Supporting Information), while a significant variation of signal to noise ratio (SNR) was recorded. The PA signal provided from the AuNP–ECFCs exhibited a strong enhancement with respect to that associated to an equivalent concentration of free AuNPs, without detectable spectral shift (Figure 4e). These results suggest that the intracellular aggregation of AuNPs leads to an increase of the efficiency of photoacoustic conversion, which may originate from a combination of the effects of electromagnetic field enhancement with the emergence of hot spots,^[^ ^34^ ^]^ and of heat and stress confinement.^[^ ^18^ ^]^ The quantification of this effect in the concentration range until 200 × 10^−6^ m is reported in Figure S2e in the Supporting Information. The evaluation of the minimum detectable amount of AuNP–ECFCs provided a value below 1 × 10^−6^ m, in terms of gold equivalent concentration in the target volume.

The photostability of the AuNP–ECFCs was also studied by prolonged laser illumination, selecting a stimulation wavelength of 860 nm, in order to get the maximum PA signal. Samples were excited over 100 s (more than 500 laser pulses). The percentage of variation among the acquired PA signals varied between 2.2% and 3.4%, the related SNR between 30 and 45, and the contrast to noise ratio (CNR) between 16 and 36 (Table S1, Supporting Information).

In order to understand the effect of particle reshaping, we studied the PA signal of AuNP–ECFCs under direct laser stimulation, without the interposition of any synthetic or biological absorber as reported in Figure S1c in the Supporting Information. We found a spectral shift of the maximum peak in the first part of NIR I optical windows from 860 to ≈700 nm, and a visible change of color in the segment of the tubes that fell under the laser beam (Figure S2d, Supporting Information).

In order to test more realistic tissue mimicking conditions, imaging was also performed under a stack of chicken breast muscle. A bolus of around 50 µL of AuNP–ECFCs 150 × 10^−6^ m was injected at different depth in samples of chicken breast tissue (Figure 4g). The PA signal generated inside the tissue was detectable until a depth of 1 cm (Figure S3, Supporting Information) with intensity in the range between 0.8 and 0.3 arbitrary units (Figure 4h,i). The PA signal produced from the AuNP–ECFCs was up to four times more intense than that originating from control regions. The image contrast was calculated for each image (using the same region of interest (ROI) dimension in a contralateral position) as reported in the Figure 4h,i, where *S* is the PA signal and *b* is the PA signal from the background.

These findings collectively provide positive premises for the use of AuNP–ECFCs for in vivo imaging.

### 4.2 Evaluation of AuNP–ECFCs Recruitment in Tumor Mass and Biodistribution Study

**Figure 5:**
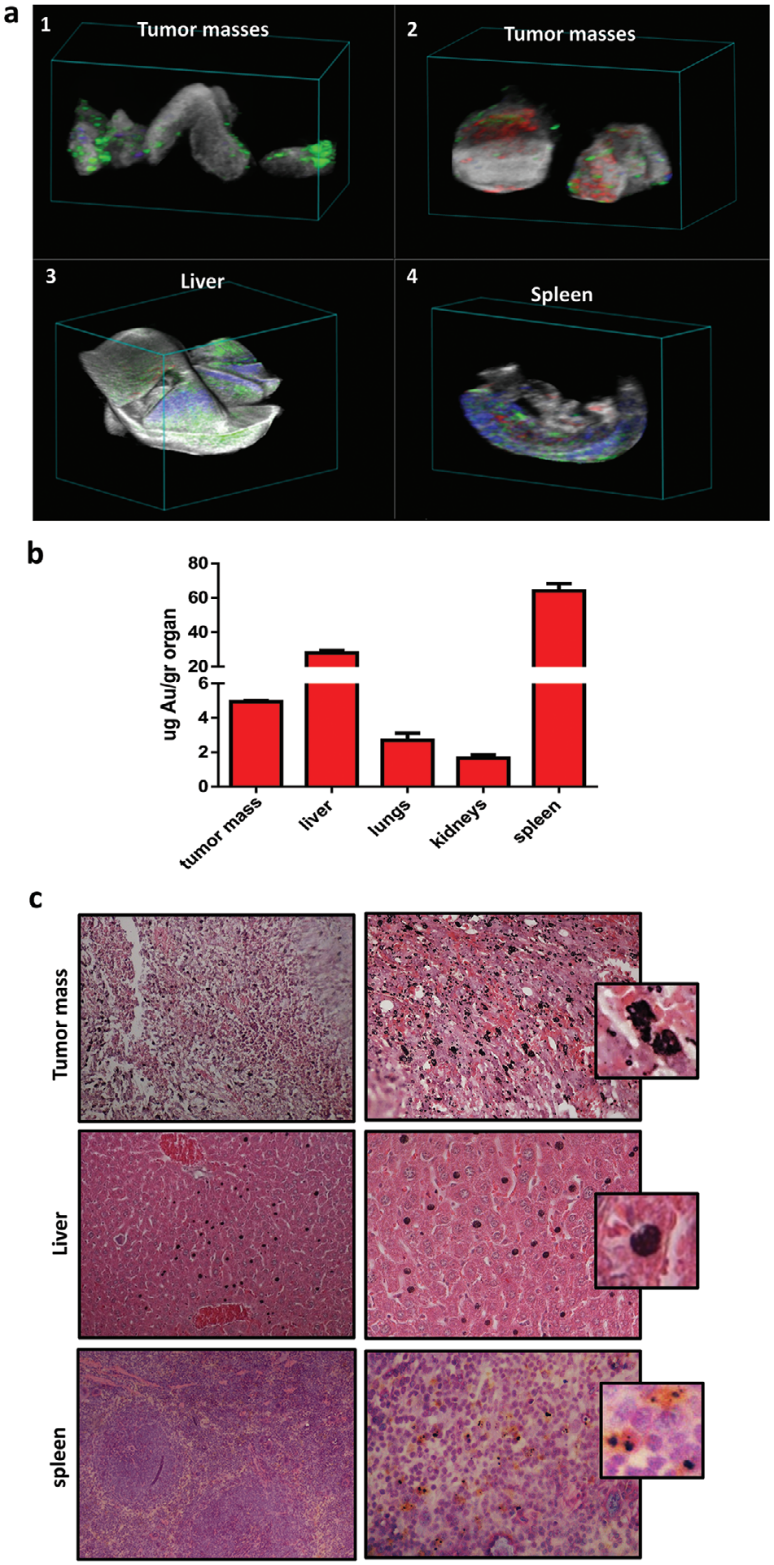
Evaluation of AuNP–ECFC migration to tumor site and of biodistribution 24 h after injection. a) 3D PA/US reconstruction of different biological targets after postprocessing spectral unmixing analysis of different compounds: 1,2) Masses of melanoma; 3) liver; 4) spleen; green scale: PA signal from the AuNP–ECFCs, red scale: PA signal from oxy‐hemoglobin, blue scale: PA signal from deoxy‐hemoglobin, and gray scale: US signal. b) Gold amount in the liver, spleen, lungs, kidney, and tumor mass 24 h after AuNP–ECFCs injection determined by ICP‐MS. c) Histological assessments of tumor tissue, liver, and spleen 24 h after AuNP–ECFC injection in the tail vein (pink: cytoplasm stained with eosin; dark blue: nuclei stained with hematoxylin).

**Figure 6:**
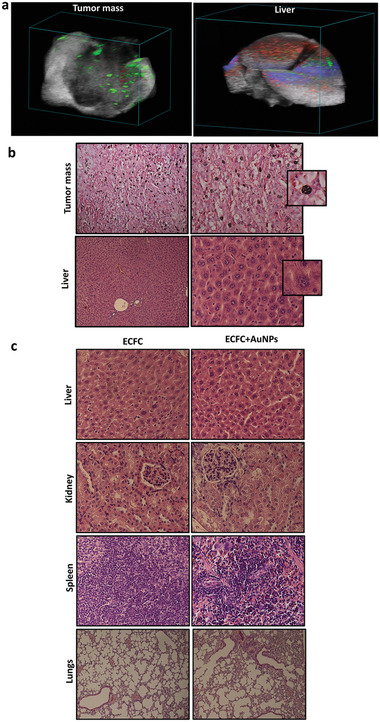
Evaluation of AuNP–ECFC migration to tumor site and of biodistribution 1 week after injection. a) 3D PA/US reconstruction of liver and mass 1 week after AuNP–ECFC injection. b) Histological section of tumor mass and liver 1 week after AuNP–ECFC administration. c) Liver, kidney, spleen, and lungs 7 days after intravenous injection of AuNP–ECFCs compared to the control group administered with unloaded ECFCs. All sections were stained with hematoxylin–eosin.

PA tests were then performed on explanted melanoma lesions and in main reticuloendothelial system organs such as the liver and the spleen, where nanoparticles are reported to preferentially accumulate. As shown in **Figure** **5**a, the internal distribution of ECFCs was assessed by postprocessing algorithms based on spectral unmixing components of each pixel based on the pure spectral signature of ground components (i.e., oxy‐ and deoxy‐hemoglobin, AuNP–ECFCs). 3D PA–ultrasound (US) visualizations and reconstructions were used to highlight global organ distributions. The endogenous components of oxy‐ and deoxy‐hemoglobin were unmixed from the spectral signal provided from the AuNP–ECFCs (Figure 5a). The 3D PA–US hyperspectral reconstruction^[^ ^35^, ^36^ ^]^ confirmed the accumulation of the AuNP–ECFCs inside the tumor as well as in the liver and the spleen. The assessment of internal distribution of ECFCs was performed by studying the target organs after sacrifice after 1 day and 1 week from injection. In the Supporting Information, we report movies of rotating and slicing 3D PA–US reconstructions, which convey a better feeling of the 2D and 3D distributions. The comparison of AuNP–ECFC accumulation in the harvested organs at 1 day (liver and spleen) and 1 week (liver) provides the evaluation of the extraction or accumulation and release of the AuNPs (Figures S4–S6, Supporting Information). We highlight in the liver that 1 week after AuNP–ECFC administration, most of the PA signal was associated to oxy‐ and deoxy‐hemoglobin (**Figure** **6**a; Figure S5, Supporting Information, red and blue colors for oxy‐ and deoxy‐hemoglobin, respectively), while after 1 day, the signal from the AuNP–ECFCs was massive (Figure 5a; Figure S4, Supporting Information)

The measurement of melanoma masses were performed by volume reconstruction (Figure S8, Supporting Information). The relative contribution of AuNP–ECFCs referred to the total amount of PA signal was typically in the range 10–30% (Table S2, Supporting Information). This findings were confirmed by ICP‐MS analysis performed in the harvested organs and tumor tissues 1 day after injection: the highest accumulation was found in the liver and spleen, followed by tumor masses (Figure 5b). Histological analyses confirmed the presence of AuNP–ECFCs^[^ ^37^ ^]^ (black spots in Figure 5c and the close‐up) in the tumor tissue, the liver but not in the spleen of mice sacrificed the day after AuNP–ECFC administration. Considering the size of the organs, AuNPs mostly accumulated in the liver, where the concentration of Au was about four times higher than in the spleen. Nevertheless, we followed the washout from these organs in 1 week: a macroscopic examination of the liver from mice sacrificed 1 week after AuNP administration revealed a lack of black spots (Figure 6b), thus confirming a significant washout of AuNPs, consistent with the massive loss of relevant PA signal (Figure 6a; Figure S5, Supporting Information). Histological analyses (Figure 6b) and PA–US renderings (Figure 6a; Figure S7, Supporting Information) demonstrate that gold is still retained in the tumor mass after 1 week from injection and that the intensity of relevant PA signal is almost comparable to that detected after 1 day (Table S2, Supporting Information). Moreover, the histological analysis of all organs including the liver, spleen, kidney, and lung did not show any morphological alteration after 7 days from administration of AuNP–ECFCs compared to organs of control mice treated with unloaded ECFCs (Figure 6c). No evidence of atrophy, hyperplasia, necrosis, and fibrosis was observed with hematoxylin–eosin staining. The examination of the parenchymal architecture did not reveal any sign of steatosis or inflammation. These results confirmed that a unique intravenous injection of 100 µg AuNPs carried by 1 × 10^6^ ECFCs efficiently reached tumors and did not induce chronic toxicity.

### 4.3 Evaluation of the In Vivo Antitumor Activity of AuNP‐Enriched ECFCs

**Figure 7:**
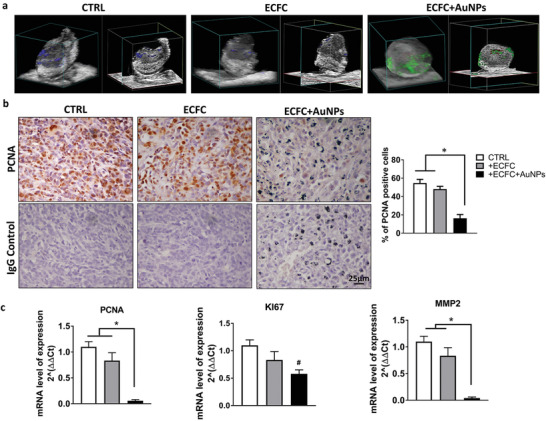
Evaluation of AuNP–ECFC antitumor activity in vivo. a) 3D US–PA volumetric reconstruction of oxygenated and deoxygenated hemoglobin PA signal distribution in the tumor masses, with step size 130 µm, and the related cross view on the right; in red and blue scales, respectively, the PA signal of oxygenated and deoxygenated hemoglobin; green scale, the PA signal of gold; gray scale for US signal. b) Top row representative PCNA stained sections of tumors from untreated mice (CTRL), ECFC, or ECFC–AUNPs injected mice. Note in the latter sample the presence of AuNPs. Bottom row representative images of sections in which the primary antibody (PCNA) was replaced by IgG of the same class. Images were acquired using 40× objective and quantified using ImageJ (NIH). Histograms representing number of PCNA stained nuclei. c) Real‐time PCR analysis performed on formalin‐fixed paraffin embedded (FFPE) melanoma tissue sections. Histograms represent relative PCNA, KI67, and MMP2‐expression levels expressed as fold change according to 2^−ΔΔCT^ method, using GAPDH gene as calibrator. Error bars: mean ± SD. Statistical analysis was performed using one‐way ANOVA followed by Newman–Keuls multiple comparison test. ^*^ *p* ≤ 0.05. # indicates significant difference from CTRL mice.

Since AuNP‐enriched ECFCs exhibited antitumor potential in vitro, we next sought to determine their antitumor activity in vivo. In the light of the reported data showing, by photoacoustic imaging and the histological analysis (Figures 6a,b and **7**a), the presence of cargo cells in the tumor mass still 1 week after injection it was conceivable to select this time point to test at the molecular level the antitumor property of AuNP–ECFCs. Animals were treated as reported in the experimental procedure and the tumor masses of the untreated mice (CTRL), ECFC‐, or AuNP‐enriched ECFCs injected mice were removed (Figure 7a) and subjected first to photoacoustic imaging to confirm the presence of gold in AuNP–ECFC‐treated mice and then processed for histological and molecular analysis. Histological analysis for proliferating cell nuclear antigen (PCNA), a known cell proliferation marker associated to the S phase of the cell cycle^[^ ^38^ ^]^ showed a strong exclusively nuclear staining in control samples (CTRL) and ECFC‐treated mice while a substantial reduction was observed in AuNP–ECFC‐treated group with only 20% of cells displaying weak nuclear staining (Figure 7b). The above observation was further supported by the expression levels of PCNA and Ki67 genes by real time PCR. Ki 67 is a ubiquitous human nuclear protein expressed in G_1_‐, S‐, and G_2_‐phases of the cell cycle but not in the G_0_‐phase^[^ ^39^, ^40^ ^]^ and is therefore a measure of the cell proliferation. A reduction of Ki‐67 gene expression and almost a complete disappearance of PCNA mRNA levels was observed for AuNP–ECFC‐treated mice. To further evaluate the antitumor activity of AuNP‐enriched ECFCs, the mRNA levels of the matrix metalloproteinase 2 (MMP‐2), which is highly expressed in melanoma and associated to tumor progression and invasion, was analyzed by real‐time PCR (Figure 7c). As shown in Figure 7c, in the ECFC–AuNPs injected mice, we observed a tenfold decrease in MMP‐2 mRNA levels compared to control mice.

### 4.4 Discussion and Conclusion

Personalized diagnosis and treatment with allogeneic or autologous cells are becoming a reality in the medical field.^[^ ^41^ ^]^ Cytotoxic or engineered T cells are under clinical trial for the treatment of hematopoietic or other malignant diseases.^[^ ^42^ ^]^ Contrast agent‐tagged macrophages are used as cellular probes to image early inflammatory processes in macrophage‐rich conditions.^[^ ^43^ ^]^ More recently, also nonimmunological cells (MSCs and endothelial progenitor cells, EPCs^[^ ^41^ ^]^) have been explored in preclinical studies of therapeutic and regenerative medicine. EPCs have been used in clinical trials for peripheral arterial disease, pulmonary hypertension, liver cirrhosis, and coronary artery disease.^[^ ^44^ ^]^ The potential therapeutic role of ECFCs, a subtype of EPCs, has been proved in ischemic disease models including myocardial ischemia, cerebral ischemia, and so on. Nonetheless, MSC and macrophages are actually the most used vectors in cell‐delivered AuNPs, thanks to their innate tumor tropism. However, a crucial issue for developing the potential of this therapeutic approach and to achieve a "tailored therapy," is the huge amount of cells either from autologous peripheral blood of cancer patients or immune privileged allogeneic cells.

Indeed, allogeneic MSCs are especially attractive due to their potential to provide immediate availability and care at the time of disease diagnosis. However, even though MSCs are believed to be immune privileged, clinical implications of immune responses to major histocompatibility complex (MHC)‐mismatched MSCs are still unknown and therefore preclinical and clinical studies are necessary to answer this critical question.

The proliferation properties of macrophages are still object of active research and a ready‐to‐use preparation for a single patient may require very long times that are not compatible with a prompt therapeutic intervention.

The largest source of EPCs is represented by umbilical cord blood (UCB), that offers immediate availability of cells with more than 450 000 unrelated units banked worldwide and available for potential clinical use.^[^ ^41^ ^]^ Nevertheless CB‐derived EPCs are relatively tolerant and display a high proliferation potential.

The current study investigate a particular subtype of CB‐ derived EPCs,^[^ ^41^ ^]^ the ECFCs, as a vehicle of AuNPs for a theranostic approach aimed at the diagnosis and thermoablative therapy of melanoma through the photoacoustic and tunable thermic properties of ECFC–AuNPs payload.

We demonstrated in vitro that AuNP‐loaded ECFCs are able to generate higher photoacoustic signals than AuNPs alone, and also display spectral fingerprints that enable a reliable detection of labeled cells following intravenous injection. We ascribe the enhancement of PA signal upon AuNP aggregation in intracellular vesicles to an interplay of electromagnetic and thermodynamic factors, such as heat and stress confinement. In vivo, we demonstrated via PA–US and ICP analysis a great tumor‐homing efficiency of AuNP–ECFCs after a bolus intravenous administration. Moreover, even though our biodistribution data showed that AuNP–ECFCs preferentially accumulate in the liver after 24 h from injection, the almost complete washout of gold from the liver after 1 week suggests an unexpected clearance. On the contrary, we proved the permanence of AuNP–ECFCs inside the tumor masses 1 week after administration. The tumor recruitment of AuNP–ECFCs is mainly due to the chemotactic gradient of SDF‐1 released in the tumor microenvironment.^[^ ^45^ ^]^ Indeed ECFCs express large amount of CXCR4, the specific receptor for the chemokine SDF‐1, on their surface. Normal tissues produce little or no SDF1 and this constitutes a strong rationale to justify the "temporary" passage of ECFCs in normal organs, followed by complete clearance within a few days. Lastly, the absence of histological damage such as fibrosis and tissue inflammation in all organs including the liver, spleen, kidney, and lung 1 week after injection, along with the healthy behavior of animals, demonstrates that AuNPs–ECFCs are safe and do not elicit any sign of toxic effects. Unambiguously, these results collectively support the advantage of ECFCs as delivery system for nanoparticle‐based diagnostic and therapeutic agents.

Another advantage of AuNP‐loaded ECFCs is the high loading efficiency that translates into a more sensitive detection of labeled cells. We also showed, for the first time, that the AuNP loading capacity of ECFCs is much greater than those of MSCs and macrophages, which have already been proposed for similar applications for their tumor tropism features.^[^ ^27^, ^28^, ^29^, ^30^ ^]^ Recent findings showed that AuNPs are able to modulate the gene expression and affect the chromatin status. We previously showed the capability of AuNPs to increase the expression levels of CXCR4 in ECFCs, and, here, we reported their effect on uPAR at mRNA and protein levels. Furthermore, we observed an increase of acetylated histone 3 that counteracts the tendency of chromatin to fold into highly compact structures, thus allowing DNA accessibility for transcriptional factors. We also demonstrated, for the first time, the antitumoral and anti‐invasive effects of ECFCs on melanoma culture with the cell cycle arrest in the G_0_–G_1_ phase and the inhibition of the collagenase activity. Nevertheless, we also observed that the effects of ECFCs on melanoma cells could shift from cytostatic to apoptotic by doubling the number of ECFCs on melanoma coculture. The in vivo data confirmed the antitumor properties of our AuNP cell‐based system by reducing the expression of cell proliferating antigens such PCNA and Ki‐67 and of the metalloproteinase MMP‐2 at the mRNA level and by inducing almost a complete disappearance of the nuclear PCNA protein. Even though Arvizo et al.^[^ ^46^ ^]^ have reported the ability of inorganic unmodified gold nanoparticles to abrogate cell tumor signaling thus leading to growth inhibition and metastasis, as far as we know, no previous research has investigated the antitumoral effects, in vitro and in vivo, of tumor tropic cells loaded with unmodified NIR AuNP without exposure to NIR light. Based on this and on our previous studies,^[^ ^32^ ^]^ ECFC‐loaded NPs 1) are phagocytosis‐prone cells with a high cytoplasm/nuclear ratio to achieve an optimal loading of nanoparticles; 2) show a marked tumor tropism and low cell retention in healthy organs; 3) represent a tracking system with simple imaging methods; 4) have an efficient thermotrasductive potential to produce locally controllable destructive energy for tumor mass without affecting normal surrounding tissues; 5) display inherent anti‐invasive and antitumor properties.

Collectively, our study represents an effort to combine multiple therapeutic options, tracking and imaging in one platform to increase the effectiveness of a single approach and taking into account that ECFCs can be easily procured and are less likely to react immunologically against the host, paves the way for future investigation as a class of theranostic agents.

## 5 Experimental Section

##### Cell Lines

ECFCs, a subpopulation of EPCs, were isolated from >50 mL human UCB of healthy newborns, as described in ref. [47] after maternal informed consent and in compliance with Italian legislation, and analyzed for the expression of surface antigens (CD45, CD34, CD31, CD105, ULEX, vWF, KDR, and uPAR) by flow‐cytometry. ECFCs were cultured in EGM2 medium (Euroclone) supplemented with 10% (v/v) fetal bovine serum (FBS, Euroclone). Cells were incubated at 37 °C with 5% CO_2_ saturation.

A375‐M6 melanoma cells (M6) were isolated in the laboratory from lung metastasis of SCID bg/bg mice i.v. injected with A375 melanoma cells. A375 cells obtained from American Type Culture Collection (Manassas, VA) and M6 cells were independently validated by STR profiling at the DNA diagnostic center BMRGenomics (Padova, Italy). Cells were amplified, stocked, and once thawed were kept in culture for a maximum of 4 months. Human MSCs were obtained from bone marrow aspirates of donors who signed informed consent, and were expanded according to published methods ref. [29]. MSCs were analyzed at P0 and P12 for the expression of surface antigens CD45, CD14, CD44, CD166, CD90, CD73, HLA‐DP, ‐DQ, ‐DR, HLA‐ABC, CD105, CD271 APC (FACSCalibur, Becton Dickinson). Cells were cultured in Dulbecco's modified Eagle's medium (DMEM, Euroclone) supplemented with 20% (v/v) fetal bovine serum (Euroclone). RAW264.7 murine macrophages were obtained from American Type Culture Collection (ATCC, Rockville, MD) and maintained at 37 °C in DMEM medium (Euroclone) in a humidified 5% CO_2_ incubator maintained at 37 °C (Thermo Scientific, Waltham, MA). Cells were grown in complete medium containing l‐glutamine and 10% inactivated fetal calf serum (Euroclone).

##### Preparation and Characterization of AuNPs

The preparation of chitosan‐capped Aumix nanoparticles (ChAumix, hereafter referred to as AuNPs) was performed according to the prescriptions in refs. [16, 32]. In order to exploit these AuNPs as fluorescent tracers, and to follow their internalization in cellular vectors, rhodamine 6G (R6G) was added. In particular, the protocols in ref. [48] were implemented, where AuNPs were conjugated to R6G and used to label ECFCs. R6G was chosen for its high fluorescence quantum yield and Raman scattering cross‐section,^[^ ^49^ ^]^ which enables a simultaneous analysis of AuNP uptake and intracellular localization.

In addition, it was verified that the AuNPs can be used as optical tracers even without secondary labels, by exploiting their photoluminescence under multiphoton excitation (see later in the description of the experimental apparatus).

Here, the main steps for the preparation of AuNPs were briefly outlined according to the recipe in ref. [16] and for their conjugation with R6G.

Tetrachloroauric(III) acid (HAuCl_4_), thiosulphate (Na_2_S_2_O_3_), and R6G were purchased from Sigma‐Aldrich and used as received. High‐molecular weight chitosan (≈106 kDa; 79% deacetylation degree) was obtained from Heppe Medical. Ultrapure water (resistivity 18 MΩ cm) was obtained from a MilliQ system from Merck Millipore.

525 µL of 3 × 10^−3^ m Na_2_S_2_O_3_ aqueous solution was rapidly added to 2.5 mL of 1.7 × 10^−3^ m HAuCl_4_ aqueous solution and vortexed for 20 s at room temperature. The molar ratio [HAuCl_4_]/[Na_2_S_2_O_3_] was adjusted to 2.7, which conveys a plasmonic band peaking around 800 nm and exhibits maximum intensity. The mixture was then left to react, and its spectrum of optical extinction was checked via UV–vis spectroscopy every 10 min until the position and shape of the plasmonic band stabilized within about 2 h. Even if the original protocol was reported to generate intrinsically stable nanoparticles,^[^ ^16^ ^]^ it was found that chitosan helped to enhance the colloidal stability of the suspension probably by the combination of electrostatic and steric effects. In addition, the net positive charge of chitosan favors the interaction between the cell membrane and the nanoparticles, thus triggering their internalization. Capping with chitosan was obtained by adding 93 µL of 10 × 10^−3^ m chitosan acidic solution (pH below 5), and letting the mixture to react for 8 h at 25 °C under gentle stirring.

The further functionalization of the AuNPs with R6G was carried out by adding 10 µL of 1 × 10^−3^ m R6G aqueous solution to 1 mL of the colloidal suspension. The mixture was stirred for 8 h and purified by three steps of centrifugation at 5500 rpm and decantation. Concentrated nanoparticles were subsequently dispersed into 1 mL water. Exposure of the final suspension to green laser light (532 nm) generated the typical orange fluorescence of R6G, thus evidencing the successful functionalization of the Au nanoparticles.

Before the biological tests, all samples were sterilized with standard autoclave treatment.

##### TEM Analysis of Au‐Enriched ECFCs

ECFCs were seeded in 6‐well plates at a density of 1.5 × 10^5^ cells per well and allowed to reach 70% confluence. Next, cells were incubated with culture medium (2 mL per well) containing suspensions of AuNPs at a concentration of 150 × 10^−6^ m Au for the indicated time points, then collected by trypsin treatment, and centrifuged at 1000 rpm for 5 min in a 1.5 mL Eppendorf tube. The cell pellet was then fixed in isotonic 4% glutaraldehyde and 1% OsO_4_, dehydrated, and embedded in Epon epoxy resin (Fluka, Buchs, Switzerland) for electron microscopy. Ultrathin sections were stained with aqueous uranyl acetate and alkaline bismuth subnitrate, and viewed and photographed under a JEM 1010 transmission electron microscope (Jeol, Tokyo, Japan) equipped with a MegaView III high‐resolution digital camera and imaging software (Jeol).

##### ICP‐AES

ECFCs cells (3.0 × 10^5^) were seeded on 10 cm dishes and allowed to attach overnight. On the next day, cells were incubated with culture medium containing AuNPs at increasing concentration, i.e., 50 × 10^−6^, 100 × 10^−6^, and 150 × 10^−6^ m Au for the indicated time points. Cells were then washed two times with PBS (Invitrogen), detached with a trypsin treatment, and counted using a hemocytometer. Cell pellets were collected by centrifugation, lyophilized, and placed in centrifuge tubes (one pellet per tube). Then, 400 µL of aqua regia was added to each tube to completely dissolve the cells and their gold content. The amount of Au was measured by Elan DRC II ICP‐MS (Perkin Elmer, Waltham, MA). For the comparative study, ECFCs, RAW264.7, and MSCs were seeded in 6‐well plates and treated for 24 h with chitosan‐coated AuNPs at a concentration of 150 × 10^−6^ m Au.

##### RNA Extraction, Semiquantitative and Quantitative PCR

Total RNA was prepared using Tri Reagent (Sigma‐Aldrich, Saint Louis, Missouri, USA), agarose gel checked for integrity, and reverse transcribed with cDNA synthesis kit (BioRad, Milano, Italy) according to the manufacturer's instructions. Selected genes were evaluated by qualitative PCR using Blue Platinum PCR Super Mix (Life Technologies, Monza, Italy) or real‐time PCR using SsoAdvanced Universal Green Mix (BioRad, Milano, Italy) with 7500 Fast Real Time PCR System (Applied Biosystems, Waltham, Massachusetts, USA). For real‐time PCR, fold change was determined by the comparative Ct method using *β*2‐Microglobulin as normalization gene. Amplification was performed with the default PCR setting: 40 cycles of 95 °C for 10 s and 60 °C for 30 s using SYBR Green‐based detection. Primer sequences (IDT, TemaRicerca, Bologna, Italy) were as follows:

18S‐rRNA: sense, 5′‐CCAGTAAGTGCGGGTCATAAG‐3′; antisense, 5′‐GCCTCACATAA‐CCATCCAATC‐3′.

uPAR: sense, 5′‐ GCCCAATCCTGGAGCTTGA‐3; antisense, 5′‐ TCCCCTTGCAGCTGTA‐ACACT‐3′.

##### Cell Sorter Analysis

To evaluate the effects of ECFCs and AuNP‐enriched ECFCs on melanoma cell proliferation, A375‐M6 cells were labeled with CFSE (Molecular Probe, LifeTtechnology) and ECFCs were labeled with Far Red (Far Red Cell Trace; Molecular Probe, Life Technologies).

In a first test, CFSE‐labeled A375‐M6 cells (CFSE‐A375‐M6) and Far Red‐stained ECFCs (FAR RED‐ECFCs) were seeded together in 10 mm petri dishes in EBM/DMEM medium at an initial density of 1 × 10^6^ and 50 × 10^4^ cells, respectively. As control, the A375‐M6 cells were seeded alone in EBM/DMEM medium at the same density. In a second test, 1 × 10^6^ CFSE‐A375‐M6 cells were cocultured with 1 × 10^5^ FAR RED‐ECFCs. After 24 h co‐incubation, A375‐M6 cells and ECFCs were harvested by trypsinization and washed once with PBS. Cells were resuspended in PBS + 0.1% Trypsin + 20 × 10^−3^ m EDTA and sorted within 1 h of harvesting. Cells were separated using BD cell sorter. CFSE‐labeled cells were detected upon excitation with a 488 nm argon laser using a 525/530 nm bandpass filter. FAR RED ECFCs were detected upon excitation at 630 nm. Cells were separately analyzed and used to establish original gating conditions. These gating limits were used as part of the sort logic. Data were acquired to 600 000 intact A375‐M6 cells and 15 000 ECFCs determined by optical scattering gating, using the acquisition software. FACS‐sorted CFSE A375‐M6 cells were used to determine the number of cells in each phase of the cell cycle based on the PI‐generated DNA histogram data.

##### Isolation of Cytoplasmic and Nuclear Fraction

1.2 × 10^6^ ECFCs were harvested and resuspended in lysis buffer (10 × 10^−3^ m HEPES pH 7.9, 50 × 10^−3^ m NaCl, 0.5 m sucrose, 0.1 × 10^−3^ m EDTA, 0.5% Triton X‐100, 1 × 10^−3^ m DTT, and a cocktail of proteinase inhibitors (Calbiochem, Merck, Darmstadt, Germany)). Samples were centrifuged at 14 000 rpm for 10 min to pellet nuclei and the cytoplasmic fraction was transferred to a new tube. Nuclei were washed twice in a buffer containing 10 × 10^−3^ m HEPES pH 7.9, 10 × 10^−3^ m KCl, 0.1 × 10^−3^ m EDTA, 0.1 × 10^−3^ m EGTA, 1 × 10^−3^ m DTT, and cocktail of proteinase inhibitors.

##### Cell Cycle Analysis

Cell‐cycle distribution was analyzed via the DNA content using the PI staining method. Cells were centrifuged and stained with a mixture of 50 µg mL^−1^ PI (Sigma‐Aldrich, St. Louis, MO, USA), 0.1% trisodium citrate, and 0.1% NP40 (or Triton X‐100) (Sigma‐Aldrich, St. Louis, MO, USA) in the dark at 4 °C for 30 min. Stained cells were analyzed via flow cytometry (BD‐FACS Canto, BD Biosciences, Franklin Lakes, NJ, USA) using the red fluorescence of propidium‐DNA.

##### Collagen Degradation Assay

A375‐M6 cell suspensions and A375‐M6 ECFC cocultures were copolymerized with Matrigel containing 2% FITC‐labeled collagen monomers (Molecular Probes). Digestion was allowed for 40 h at 37 °C and solid‐phase Matrigel containing the cells was pelleted, whereas FITC released into the supernatant was analyzed by spectrofluorometry. The 100% reference was obtained by complete collagenase digestion of cell‐free Matrigel lattices. Background fluorescence was obtained by pelleting nondigested cell‐free FITC collagen‐enriched Matrigel layers.

##### Western Blot Analysis

Harvested cells were resuspended in 20 × 10^−3^ m RIPA buffer (pH 7.4) (Merk Millipore, Vimodrone, MI, Italy) containing a cocktail of proteinase inhibitors (Calbiochem, Merck, Darmstadt, Germany) and treated by sonication (Microson XL‐2000, Minisonix, Farmingdale, NY, USA).

Aliquots of supernatants containing equal amounts of protein (30 µg) in Laemmli buffer were separated on Bolt Bis‐Tris Plus 4–12% precast polyacrylamide gels (Life Technologies, Monza, Italy). Fractionated proteins were transferred from the gel to a PVDF nitrocellulose membrane using an iBlot 2 system (Life Technologies, Monza, Italy). Blots were stained with Ponceau red to ensure equal loading and complete transfer of proteins, and then blocked for 1 h at room temperature with 5% milk in PBS containing 0.1% Tween. Subsequently, membranes were probed at 4 °C overnight with mouse anti‐uPAR antibody (1:500 Mon R4; ThermoFisher) or rabbit anti‐*α*‐tubulin antibody (1:1000 Cell Signaling) used to assess equal amounts of protein loaded in each lane. Antirabbit IgG (whole molecule)–Peroxidase antibody (Sigma, Cat#A0545) or antimouse IgG (whole molecule)–Peroxidase antibody (Sigma, Cat#A9044) were used as secondary antibodies; the enhanced chemiluminescence (ECL) procedure was employed for development.

##### Confocal Microscopy Analysis

Cells were grown on glass coverslips, washed twice with 1 mL of PBS, fixed for 20 min in 3.7% paraformaldehyde in PBS, and permeabilized with 0.1% Triton X‐100 in PBS for 5 min. Cells were incubated in blocking buffer (3% BSA and 0.1% Triton X‐100 in PBS) for 1 h at room temperature and then stained with phalloidin for 1 h. Nuclei were stained with fluorescent Hoechst 33342 dye (DAPI) (10 µg mL^−1^) (Invitrogen) for 15 min at RT. The coverslips containing the labeled cells were mounted with an antifade mounting medium (Biomeda, Foster City, CA) and observed under a Bio‐Rad MRC 1024 ES Confocal Laser Scanning Microscope (Bio‐Rad, Hercules, CA) equipped with a 15 mW Krypton/Argon laser source for fluorescence measurements. Cells were examined with a Nikon Plan Apo X60‐oil immersion objective using an excitation wavelength appropriate for Alexa 488 (495 nm). Series of optical sections (XY: 512 × 512 pixels) were then taken through the depth of the cells with a thickness of 1 µm at intervals of 0.8 µm (Z step). A single composite image was obtained by the superimposition of 20 optical sections for each sample.

##### MPL Analysis

As anticipated, the use of AuNPs was explored as multiphoton luminescence emitters^[^ ^50^, ^51^ ^]^ to infer information on their cellular uptake, as a confirmation of the results obtained with R6G‐marked AuNPs.

The multiphoton laser scanning confocal microscope was based on a Zeiss LSM 510 Meta NLO system equipped with a Coherent Chameleon Ti:Sapphire laser coupled to an upright Zeiss Axioskop 2 microscope (Carl Zeiss Microscopy, Thornwood, NY). The ECFCs labeled with AuNPs (incubation condition: 100 × 10^−6^ or 150 × 10^−6^ m Au equivalent for 24 h) were seeded onto sterile cover glasses, fixed using 3.7 vol% formaldehyde in PBS, and subjected to multiphoton imaging at an excitation wavelength of 800 nm. The photoluminescence intensity of the cells at each time point was obtained by averaging over at least 10 cells from multiple images. The laser power at different time points did not show variations larger than 1%.

##### Photoacoustic Imaging In Vitro and Ex Vivo

PA experiment was performed using the multimodality imaging platform Vevo LAZR by FUJIFILM Visualsonics Inc. (Toronto). The PA properties and performances of ECFCs were evaluated in test‐object phantoms and ex vivo samples of biological tissue. The test‐object PA characterization was performed using a custom made phantom (Figure 4a) consisting of a polypropylene box loaded with coplanar PE tubes (internal diameter = 0.58 mm; external diameter = 0.99 mm) that were loaded with AuNP–ECFCs at different concentrations (Figure 4b) in the range of 12 × 10^−6^ to 200 × 10^−6^ m. The strong optical absorbance of laser pulses from the AuNPs that originates the PA emission may also trigger their photoinstability, due to overheating and reshaping.^[^ ^52^ ^]^ In order to prevent this nuisance, the optical fluence reaching the AuNPs was limited by the interposition of a thin layer of chicken breast placed over the PE tubes. Figures 5a and 6a report the 3D PA/US reconstructions (38 µm motor‐step) of this experimental configuration, where the green‐scale encoded the intensity of PA signal generated from the AuNP–ECFCs, and the grayscale the co‐registered US signal from the PE tubes and the biological tissue. A second data set was obtained after injection of a bolus of around 50 µL of AuNP–ECFCs within the chicken breast, which served as reference biological matrix.^[^ ^53^ ^]^

Ex vivo tests were performed on harvested tissues of melanoma, liver, and spleen of the mouse models, post‐AuNP–ECFC treatment at time points of 1 day and 1 week.

The PA multispectral analysis was performed in the first optical window from 680 to 970 nm, in order to identify their specific fingerprint. The photostability was evaluated by keeping the AuNP–ECFCs under prolonged laser illumination over time at fixed wavelength, and then calculating the CNR, the SNR, and the percent coefficient of variation (%CV) as

(1) $$\mathrm{CNR}=\frac{\;S-b}{\sqrt{\sigma_{s}^{2}+\sigma_{b}^{2}}}\;\mathrm{SNR}=\frac{S}{\sigma_{S}}\;\%\mathrm{CV}=100\cdot \frac{\sigma_{S}}{S}$$

where *S* is the acquired PA signal, *b* is the background, and *σ* is the standard deviation.

A comparative PA test was performed between the AuNP–ECFCs and the AuNPs alone, in order to test the hypothesis of a differential PA effect produced when the AuNPs aggregate upon internalization in the ECFCs. Moreover, 3D PA–US visualizations of harvested organs were reconstructed to understand the AuNP–ECFC organ distribution and to assess the global amount of PA signal. Spectral unmixing techniques were applied to highlight the PA signal provided from the AuNP–ECFCs with respect to that from endogenous dyes, such as oxy‐ and deoxy‐hemoglobin.^[^ ^54^ ^]^

##### In Vivo Experiments

The procedures involving animals were performed in accordance with the ethical standards, the Declaration of Helsinki and national guidelines, approved by the ethical committee of Animal Welfare Office of Italian Work Ministry (protocol # 401/2015) and conformed to the legal mandates and Italian guidelines for the care and maintenance of laboratory animals.

Fourteen CD1 immunodeficient mice (6–8 weeks old; Charles River Laboratories International) were injected subcutaneously with 100 µL of PBS containing 1 × 10^6^ M6 cells. On day 14, when tumor measured ≈150 mm^3^, one group of ten animals was injected intravenously with ECFCs preincubated with AuNPs at a concentration of 150 × 10^−6^ m Au, and four mice were injected with unloaded ECFCs. Animals were sacrificed at different times after ECFC injection: one group of eight AuNP–ECFC animals and one group of two unloaded ECFC mice (Control mice) were sacrificed 24 h later, and one group of two AuNP–ECFC mice and another of two control mice after 1 week.

Mice were anesthetized with a solution of ketamine (0.75 mg kg^−1^ body weight) and xylazine (0.10 mg kg^−1^ body weight), and harvested samples were fixed in 4% buffered formalin and directed to ex vivo PA analysis, histological examination, and assessment of gold concentration.

The histological analysis of formalin fixed tumor mass and organs was performed on paraffin‐embedded sections (4 µm) using hematoxylin and eosin. Stained slides were examined using an optical microscope.

To quantify the amount of gold in the tumor and organs, resected tissues were prepared and analyzed using ICP‐MS. Samples were frozen in liquid nitrogen and weighed, then digested in aqua regia, and prepared as previously described.^[^ ^32^ ^]^

To evaluate the antitumor effect of ECFC‐enriched AuNPs in vivo, 12 male CD1 immunodeficient mice (6–8 weeks old; Charles River Laboratories International) were injected subcutaneously with 100 µL of PBS containing 1 × 10^6^ M6 cells. On day 14, when tumor measured ≈150 mm^3^, one group of four animals was injected intravenously with PBS (CTRL), four animals with ECFCs preincubated with AuNPs at a concentration of 150 × 10^−6^ m Au (ECFCs+AuNPs), and four mice were injected with unloaded ECFCs (ECFCs). One week after the injection mice were sacrificed, the tumor mass was removed and fixed overnight at 4 °C in formalin (5% in PBS). Histological and molecular analysis were performed on paraffin embedded sections.

##### Immunohistochemistry

4 µm thick paraffin‐embedded tumor sections were collected on glass slides. After deparaffinization, through a series of solutions (100% xylene through 100% ethanol to 100% water), slides were boiled for antigen retrieval and treated with 3% H_2_O_2_. The slides were subsequently blocked with 1.5% BSA and incubated with PCNA primary antibody (1:200, Mouse mAb #2586 Cell Signaling) overnight at 4 °C. Probed slides were then subjected to biotinylated mouse (1:1000, Vector Laboratories, Inc., Burlingame, CA, USA) secondary antibody. The antibody binding activity was detected using the avidin–biotin–peroxidase complex method and diaminobenzidine tetrahydrochloride chromogen kit (Dako LSAB2; Dako Corporation, Carpinteria, CA). Slides were counterstained with aqueous Meyer hematoxylin and mounted with glycerol for visual inspection and photography. For negative control in the IHC procedures, primary antibody was replaced by IgG of the same class.

##### Formalin‐Fixed Paraffin‐Embedded (FFPE) RNA Extraction Sample and Quantitative RT‐PCR (qRT‐PCR)

Twelve 10 µm thick sections were cut from each block of FFPE tissue, transferred to 1.5 mL sterile tubes, and processed using the PureLink FFPE Total RNA Isolation Kit (Invitrogen, by Thermofisher) as previously described.^[^ ^55^ ^]^ Briefly, RNA was extracted by spin column purification according to similar basic principles: deparaffinization, followed by cell disruption with heated proteinase K, which is capable of efficiently degrading proteins that were covalently crosslinked with each other and RNA. Proteinase K incubation at high temperature (60 to 70 °C) also removes part of the methylol additions induced by formalin fixation. After proteinase K incubation, RNA was isolated by alcohol precipitation in a spin column purification step and then was stored at −80 °C. Total RNA 260/280 OD ratios were consistently between 1.7 and 1.85, indicating high sample purity.

500 ng RNA was reverse‐transcribed using Thermo Scientific Maxima H Minus cDNA Synthesis Master Mix with dsDNase (Invitrogen, by Thermofisher) according to manufacturer's instructions.

Real‐time RT‐PCR was performed using ABI PRISM 7500 Sequence Detection System instrument and software (Applied Biosystems, USA). The relative expression level of the house‐keeping gene (18S‐rRNA) and three target genes (Ki67; PCNA and MMP‐2) was measured using SYBR Green dye‐based method. Relative mRNA expression of a target gene within a specimen was calculated as 2^−ΔCT^, where Δ*C*_T_ = *C*_T(target gene)_ − *C*_T(housekeeping gene)_. The primer sequences were as follows:

Human Ki67: forward 5′‐TCCTTTGGTGGGCACCTAAGACCTG‐3′ and reverse 5′‐TGATGGTTGAGGTCGTTCCTTGATG‐3′.
Human PCNA: forward 5′‐TCCTCCTTCCCGCCTGCCTGTAGC‐3′ and reverse 5′‐CGCGTTATCTTCGGCCCTTAGTGTA‐3′.
Human MMP‐2 forward 5′‐CCCCAAAACGGACAAAGAG‐3′ and reverse 5′‐CACGAGCAAAGGCATCATCC‐3′.
Human 18S‐rRNA: sense,5′‐CCAGTAAGTGCGGGTCATAAG‐3′; antisense, 5′‐GCCTCACATAA‐CCATCCAATC‐3′.

##### Statistical Analysis

All the in vitro results were reported as means with standard deviation (SD). For comparisons of more than two groups, a one‐way analysis of variance (ANOVA) followed by Newman–Keuls post test was used. When only two groups were compared, statistical significance was assessed with an unpaired Student's *t*‐test. *p* values of less than 0.05 were considered significant. Data were analyzed using GraphPad Prism 6. The statistical tests used are stated in the figure captions. The elaboration of the PA data was conducted using the following software: VevoLab (Fujifilm Visualsonic Inc., Toronto) and Originlab. The statistical analysis to check and compare the different kind of data, spectral acquisitions, and photostability over time under prolonged laser illumination was done by one‐way ANOVA test with *p* ≤ 0.05 level of significance. The PA signal values acquired during the spectral analysis were carried out in the optical windows from 680 to 970 nm with a step size of 5 nm. All of these PA values, for each concentration, were determined on three different set of data, and then the mean value for each one wavelength and its standard deviation was calculated. Finally, using one‐way ANOVA (*p* ≤ 0.05), the other set of spectral data, provided from the other colloidal solution at different concentrations, was analyzed and compared in order to underline their PA intensity differences correlated with the concentrations. The data set concerning the photostability was acquired over 100 s, 5 values per second, for an amount of over 500 points. Also, these datasets were compared in the same way, calculating the PA signal mean values and the related standard variation, and verifying by one‐way ANOVA test (*p* ≤ 0.05), matching the goodness of the results for the datasets at the same concentration (not significantly different) and those at different concentrations (significant differences). All the statistical tests confirmed the right match with the hypothesis, in terms of spectral trends and the performing photostability. All regarding biomedical imaging elaborations and analysis were done by the Vevolab software. The Vevolab had allowed to check the different regions of acquired PA images in order to find the spectral trends and to discriminate the different PA spectral trends provided from the AuNP‐enriched ECFCs and the endogenous responsive molecules (oxygenated and deoxygenated hemoglobin) inside the treated samples. The spectral unmixing tool operated on 2D and 3D acquisitions, in particular, the 3D calculations for spectral discrimination were made on slice of 130 µm thickness on sample volumes with dimensions of the order of 1.5 cm. The PA spectral trends were calculated and evaluated on 59 points, while the photostability was checked and evaluated shooting the samples for over 500 laser shots.

## Conflict of Interest

The authors declare no conflict of interest.

## Author Contributions

G.F. and A.L. contributed equally to this work. A.L., M.D.R., and G.F. conceived and designed the study. P.A. and L.M. performed and designed the photoacoustic experiments. F.R., S.C., G.M., and T.D.R. synthetized and characterized AuNPs. F.M., A.C., and A.B. performed molecular analysis. F.B. performed animal experiments, and E.R. carried out cytological and histological analysis. M.L. performed cell sorting and FACS analysis. D.B. carried out TEM analysis. A.M., A.C., and A.B. analyzed and interpreted the data. R.T. and M.S. performed the ICP analysis on cells and biological tissues. G.F., M.D.R., and A.L. wrote and revised the manuscript. M.D.R. obtained funding.

## Supporting information

Supporting Information

Supplemental Movie 1

Supplemental Movie 2

Supplemental Movie 3

Supplemental Movie 4

Supplemental Movie 5
