# Supplementary material for: Enhanced Antitumoral Activity and Photoacoustic Imaging Properties of AuNP‐Enriched Endothelial Colony Forming Cells on Melanoma
Source: Adv Sci (Weinh). 2020 Dec 21;8(4):2001175. doi: 10.1002/advs.202001175 (PMC7887578; doi:10.1002/advs.202001175)
Supplement: Supplementary file 1 — Supporting Information [file ADVS-8-2001175-s001.pdf]

© 2020 Wiley-VCH GmbH

ADVANCED SCIENCE
Open Access

Supporting Information

for *Adv. Sci.,* DOI: 10.1002/advs.202001175

Enhanced Anti-tumoral Activity and Photoacoustic Imaging Properties of AuNP-Enriched Endothelial Colony Forming Cells on Melanoma Cells

Paolo Armanetti, Anastasia Chillà, Francesca Margheri, Alessio Biagioni, Luca Menichetti, Giancarlo Margheri, Fulvio Ratto, Sonia Centi, Francesca Bianchini, Mirko Severi, Rita Traversi, Daniele Bani, Matteo Lulli, Tommaso Del Rosso, , Alessandra Mocali, Elisabetta Rovida, Mario Del Rosso, Gabriella Fibbi,* and Anna Laurenzana,**

Supplementary information (SI)

Title: "Enhanced anti-tumoral activity and photoacoustic imaging properties of AuNP-enriched Endothelial Colony Forming Cells on melanoma cells"

Paolo Armanetti[1], Anastasia Chillà[2], Francesca Margheri[2], Alessio Biagioni[2], Luca Menichetti[1], Giancarlo Margheri[3], Fulvio Ratto[4], Sonia Centi[4], Francesca Bianchini[2], Mirko Severi[5], Rita Traversi[5] , Daniele Bani[6], Matteo Lulli[2] , Tommaso Del Rosso[7], , Alessandra Mocali[2], Elisabetta Rovida[2], Mario Del Rosso[2], Gabriella Fibbi[2*†], Anna Laurenzana[2*†]*

[1] *Institute of Clinical Physiology (IFC), National Research Council Pisa, Italy*
[2] *Department of Experimental and Clinical Biomedical Sciences, University of Florence,*
[3] *Institute for Complex Systems, National Research Council, Sesto Fiorentino, Italy*
[4] *Institute of Applied Physics 'N. Carrara', National Research Council, Sesto Fiorentino, Italy*
[5] *Department of Chemistry "Ugo Schiff", University of Florence, 50019 Sesto Fiorentino, Italy*
[6] *Department of Clinical and Experimental Medicine, University of Florence, 50134, Florence Italy*
[7]*Department of Physics, Pontifícia Universidade Católica do Rio de Janeiro, Brazil*

†These authors contributed equally to this paper.

* Corresponding Authors :

Dr. L. Menichetti, email: luca.m@ifc.cnr.it
Dr. G. Fibbi, email: fibbi@unifi.it
Dr. A. Laurenzana, e-mail: anna.laurenzana@unifi.it,

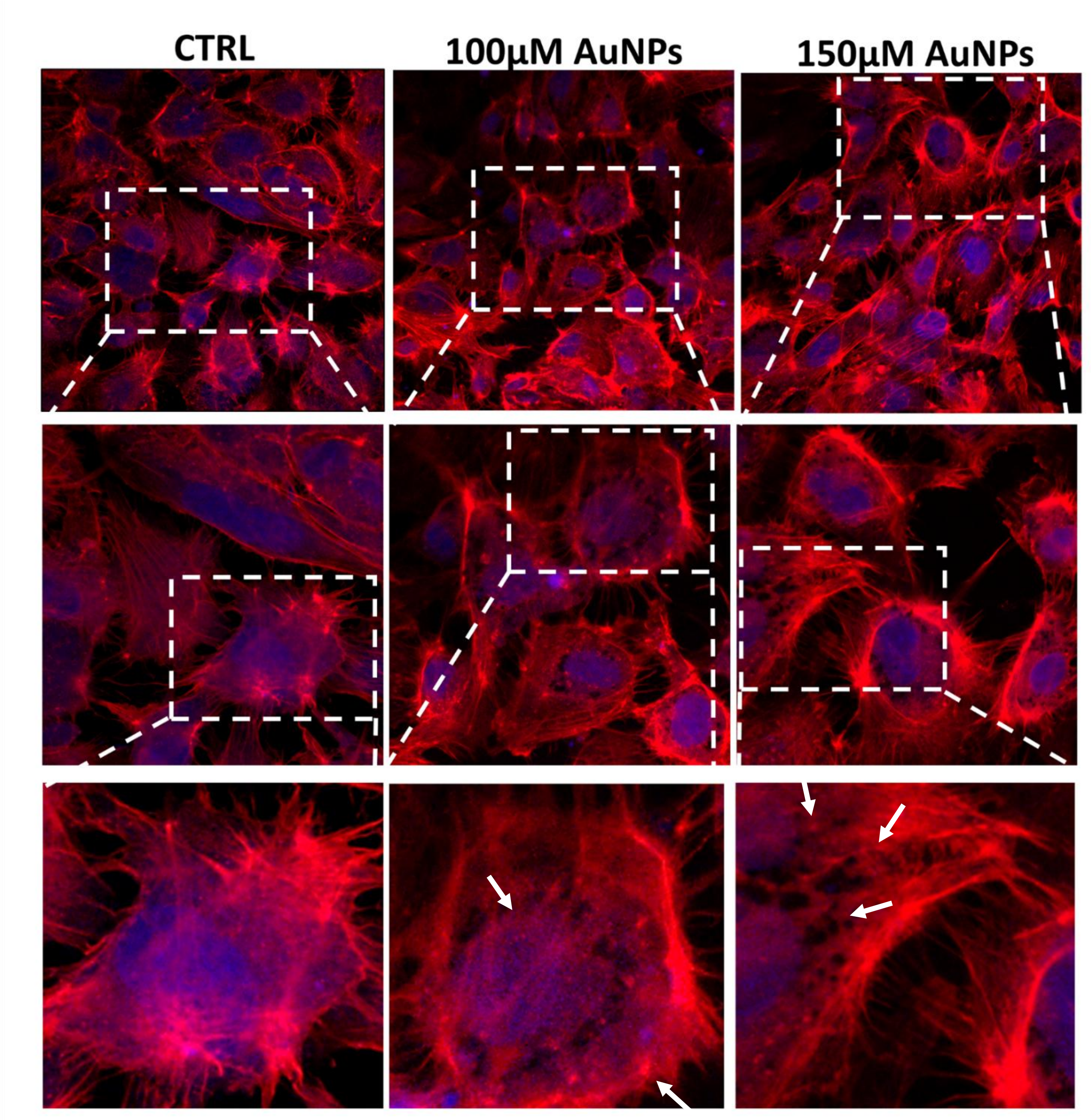

Supplementary Figure 1:

Fluorescence microscopy of untreated (CTRL) or treated ECFCs with increasing dose of AuNPs and labelled with cytoskeletal F-Actin staining phalloidin. AuNPs accumulated and formed intracellular aggregates (black spots pointed by white arrows) in perinuclear areas clearly visible in the magnified selected areas.

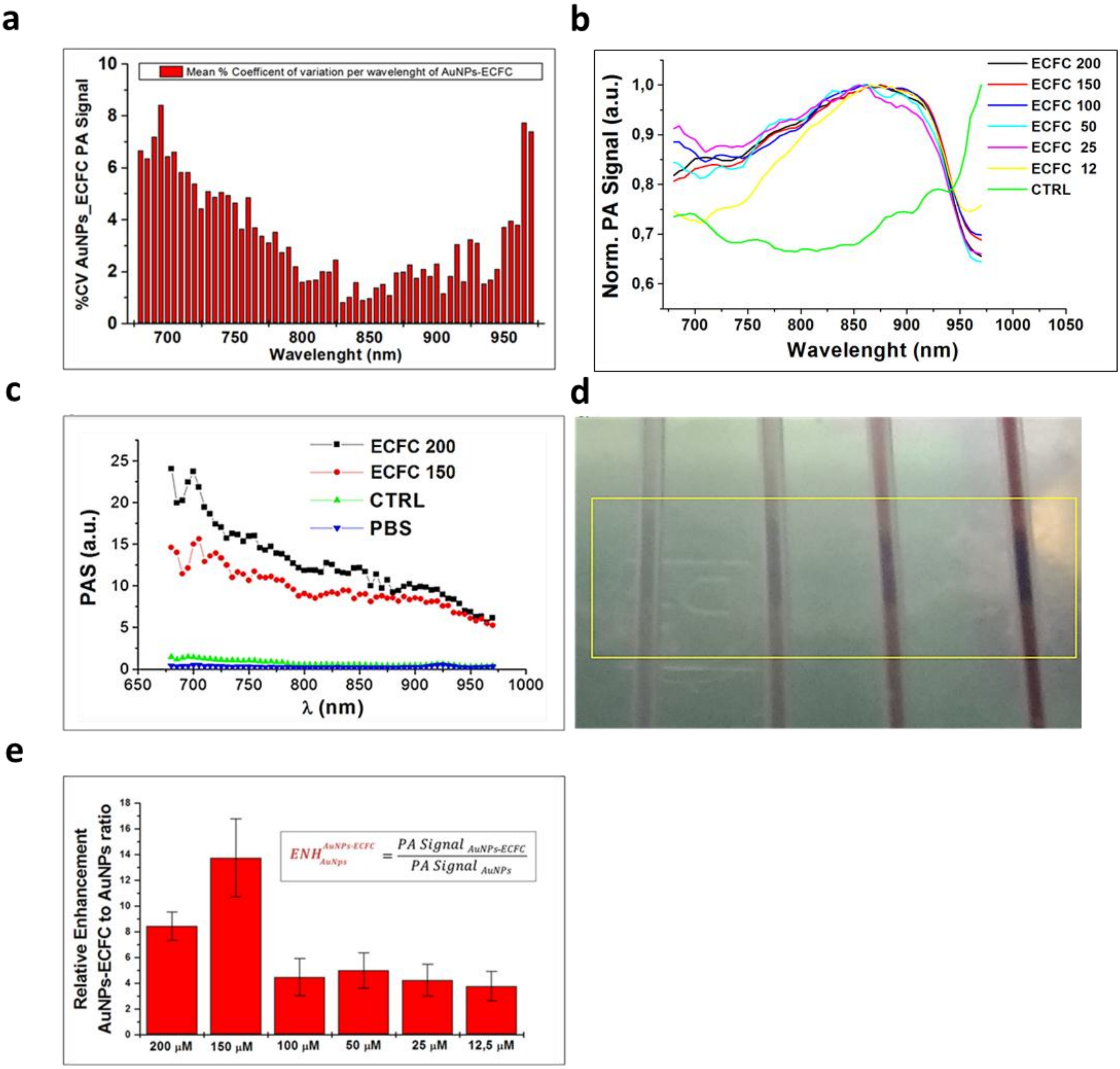

Supplementary Figure 2:

PA imaging of AuNP-ECFC in test-objects

a) Mean coefficient of variation of AuNP-ECFCs at the different laser wavelength stimulation;

b) Plot of the photoacoustic signals produced by ECFC at different concentration of internalized nanoparticles in terms of Au concentration (200, 150, 100, 50, 25, 12 µM, black to yellow plots) and the normalized photoacoustic signal provided from the control, in which we utilized the unloaded ECFC (green plot);

c) PA spectral trend of AuNP-ECFCs under direct pulsed laser illumination, the of AuNP-ECFCs got a shift around 700 nm, that's means the modification of the longitudinal PSR of AuNPs due to the change of the nano-structure geometry due to reshaping phenomena;

d) the picture of three sections of PE tubes loaded with the AuNP-ECFCs (50, 100, 150 and 200 µM) in which the AuNP-ECFCs got reshaping;

e) Graphic of the ratio between the intensity of PA signal provided from AuNP-ECFCs respect that of AuNPs, calculated by the shown formulation, to quantify the enhancement of the PA signal of AuNPs when loaded into the cells.

**AuNP-ECFC Au (µM)**	**PA Signal (a.u.)**	**Std.Dev.**	**%CV**	**SNR**	CNR
**200**	1,863	0.051	2,711	36,884	32,592
**150**	1,171	0.025	2,175	45,981	36,225
**100**	0,694	0.022	3,160	31,644	21,207
**50**	0,610	0.018	2,882	34,693	21,168
**25**	0,565	0.016	2,892	34,583	20,005
**12**	0,499	0,017	3,309	30,220	16,368
**CTRL**	0,190	0,009	4,882	20,484	

Table SI 1: Photostability test under prolonged laser stimulation with fixed wavelength at the localized surface plasmon resonance (LPSR) peak: photoacoustic mean values from tubes loaded at different AuNP-ECFC concentrations, standard deviation, percentage variation coefficient (CV), contrast to noise ratio (CNR), signal to noise ratio (SNR)

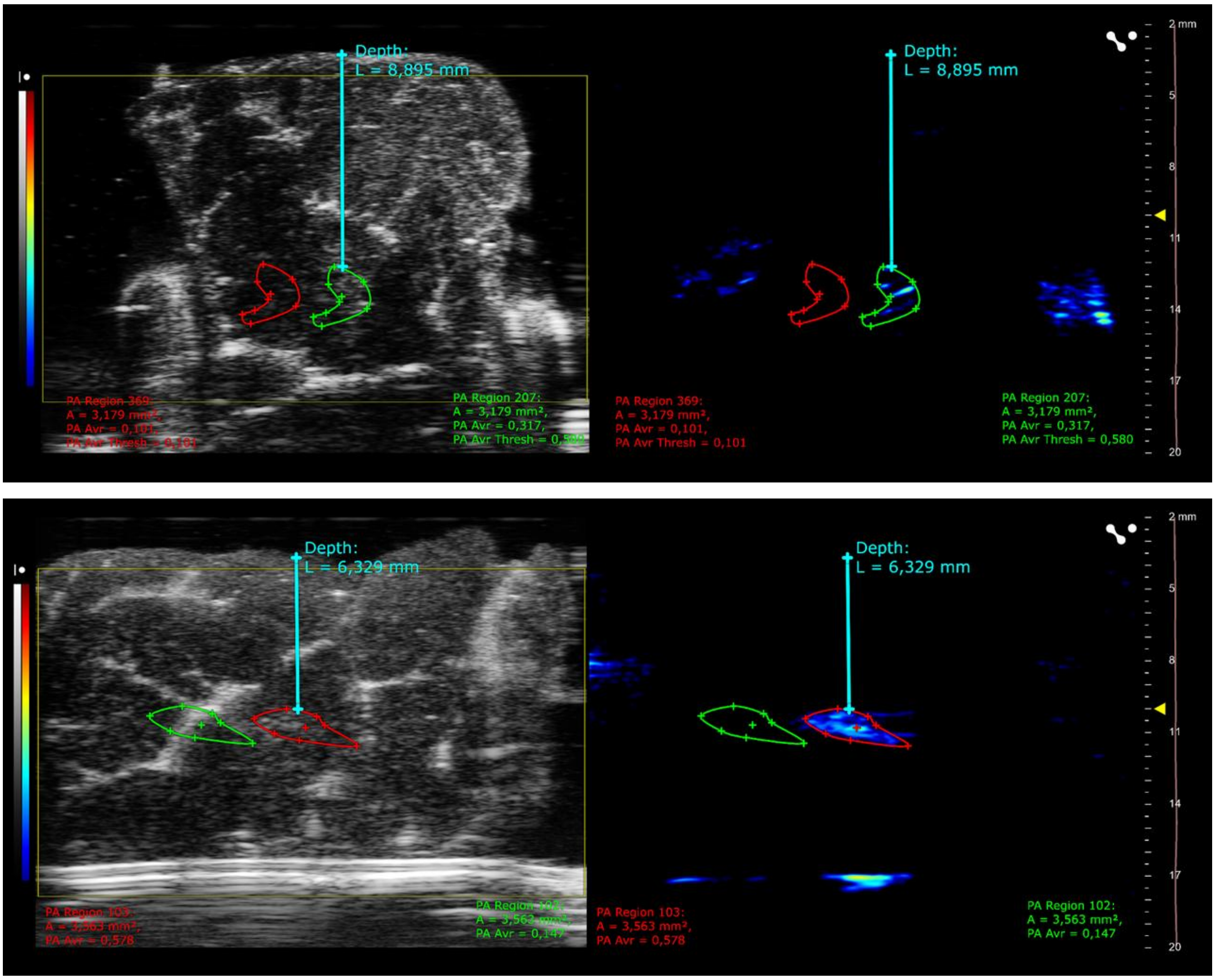

Supplementary Figure 3

PA imaging of AuNP-ECFC in test objects

Samples of chicken breast injected with a bolus of around 50 µL of AuNP-ECFC and the calculation of PA signal depth, in colored scalebar the intensity of PA signal, in the graybar the intensity of ultrasonic signal.

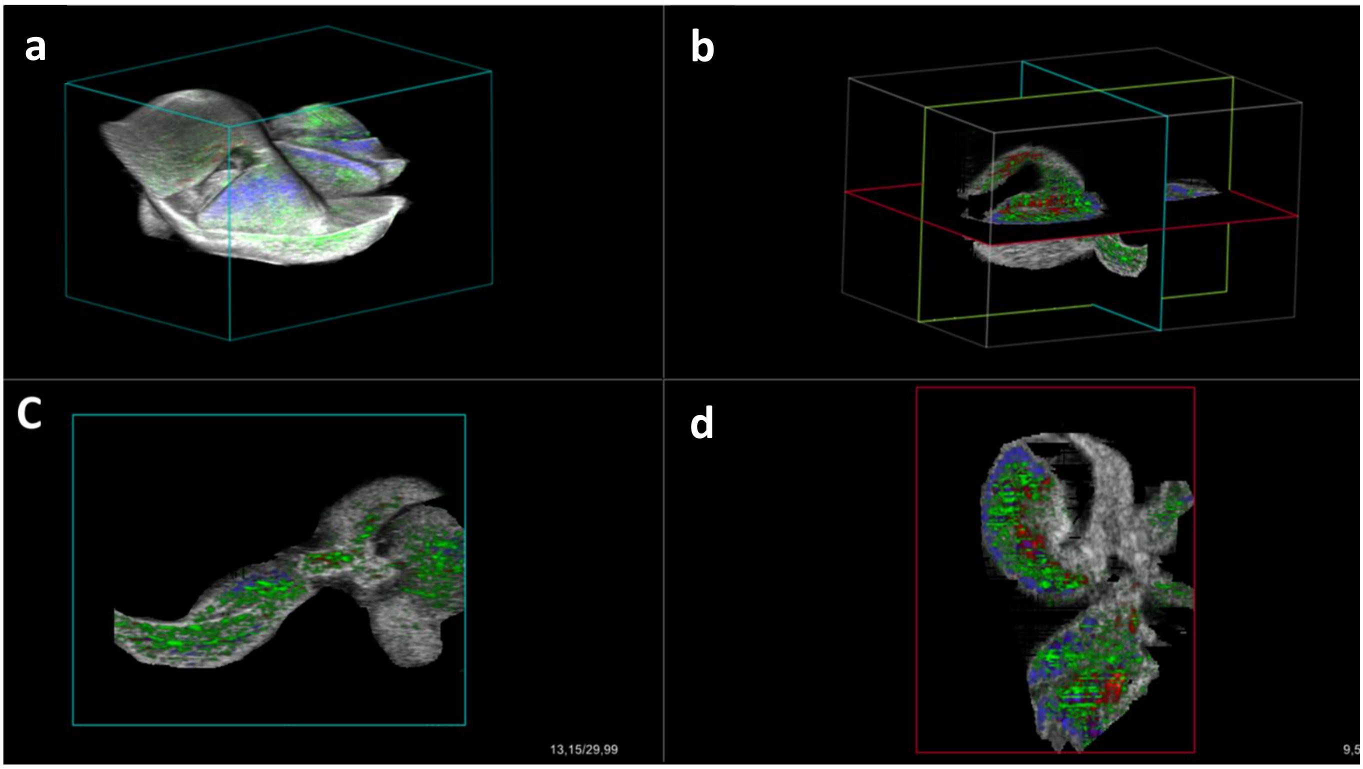

Supplementary Figure 4

3D PAUS rendering of liver one day after AuNP-ECFC treatment, images acquired with 120 µm 3D motor-step and analyzed in post-processing by spectral unmixing algorithm

(a) whole 3D liver volume;

(b) 3D texture mapping of three section of interest inside the liver;

(c) maximum signal intensity sagittal slice view of liver;

(d) maximum signal intensity transverse slice view of liver; in green color the PA signal from AuNP-ECFCs, in red color the PA signal of oxygenated hemoglobin, in blue color the PA signal of deoxygenated hemoglobin.

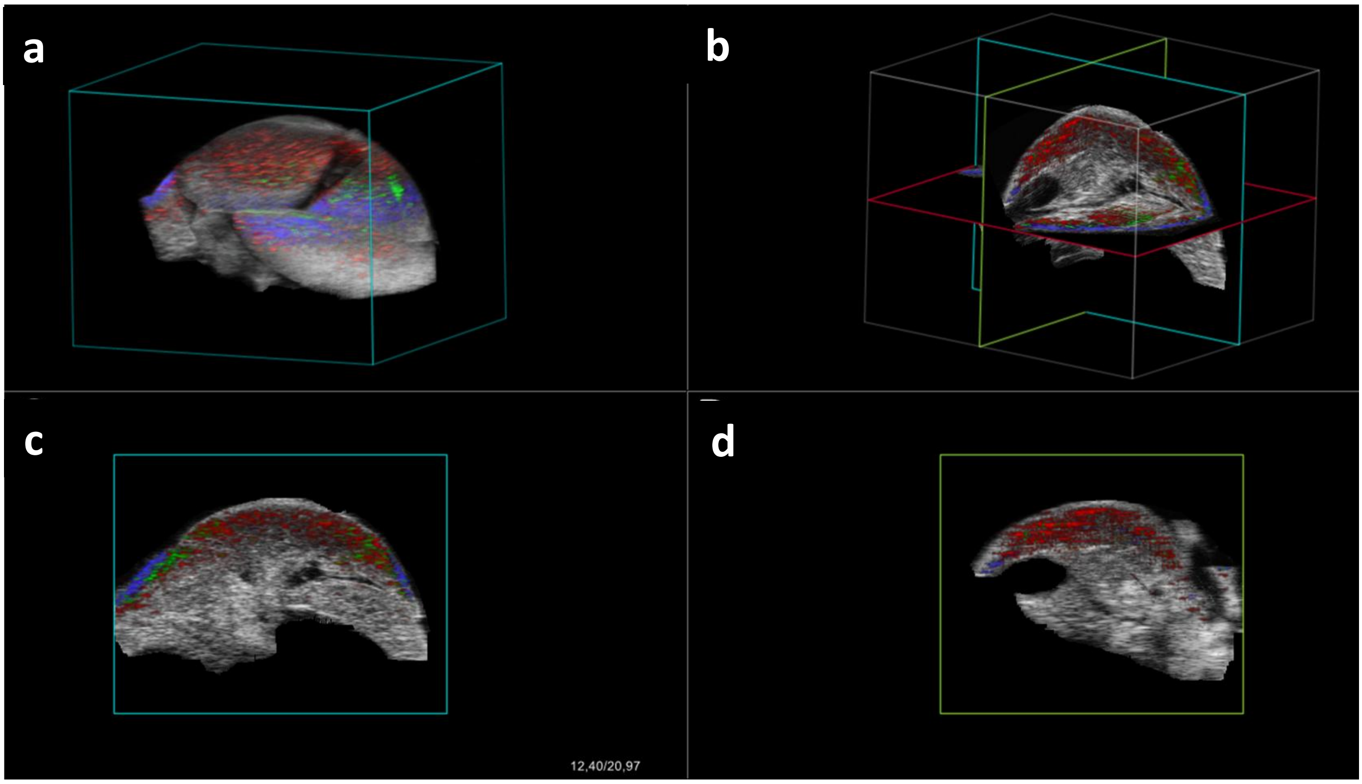

Supplementary Figure 5

3D PAUS Volume reconstruction of liver one week after AuNP-ECFC treatment, images acquired with 120 µm 3D motor-step and analyzed in post-processing by spectral unmixing algorithms

(a) whole 3D liver volume;

(b) 3D texture mapping of three section of interest inside the liver;

(c) maximum signal intensity sagittal slice view of liver;

(d) maximum signal intensity coronal slice view of liver; in green color the PA signal from AuNP-ECFCs, in red color the PA signal of oxygenated hemoglobin, in blue color the PA signal of deoxygenated hemoglobin.

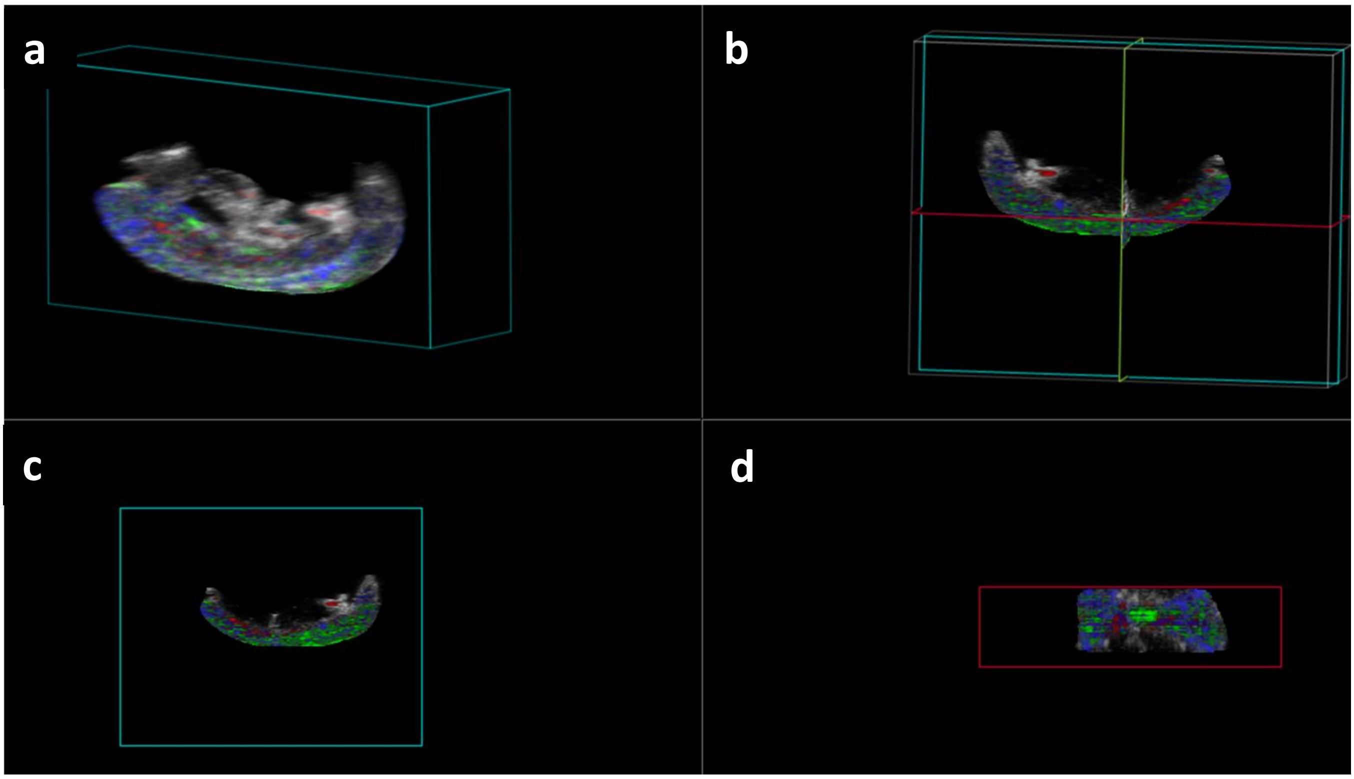

Supplementary Figure 6

3D PAUS Volume reconstruction of spleen one day after AuNP-ECFC treatment, images acquired with 120 µm 3D motor-step and analyzed in post-processing by spectral unmixing algorithm3D

(a) whole 3D spleen volume;

(b) 3D texture mapping of three section of interest inside the spleen;

(c) maximum signal intensity sagittal slice view of spleen;

(d) maximum signal intensity transverse slice view of spleen; in green color the PA signal from AuNPs- ECFC, in red color the PA signal of oxygenated hemoglobin, in blue color the PA signal of deoxygenated hemoglobin.

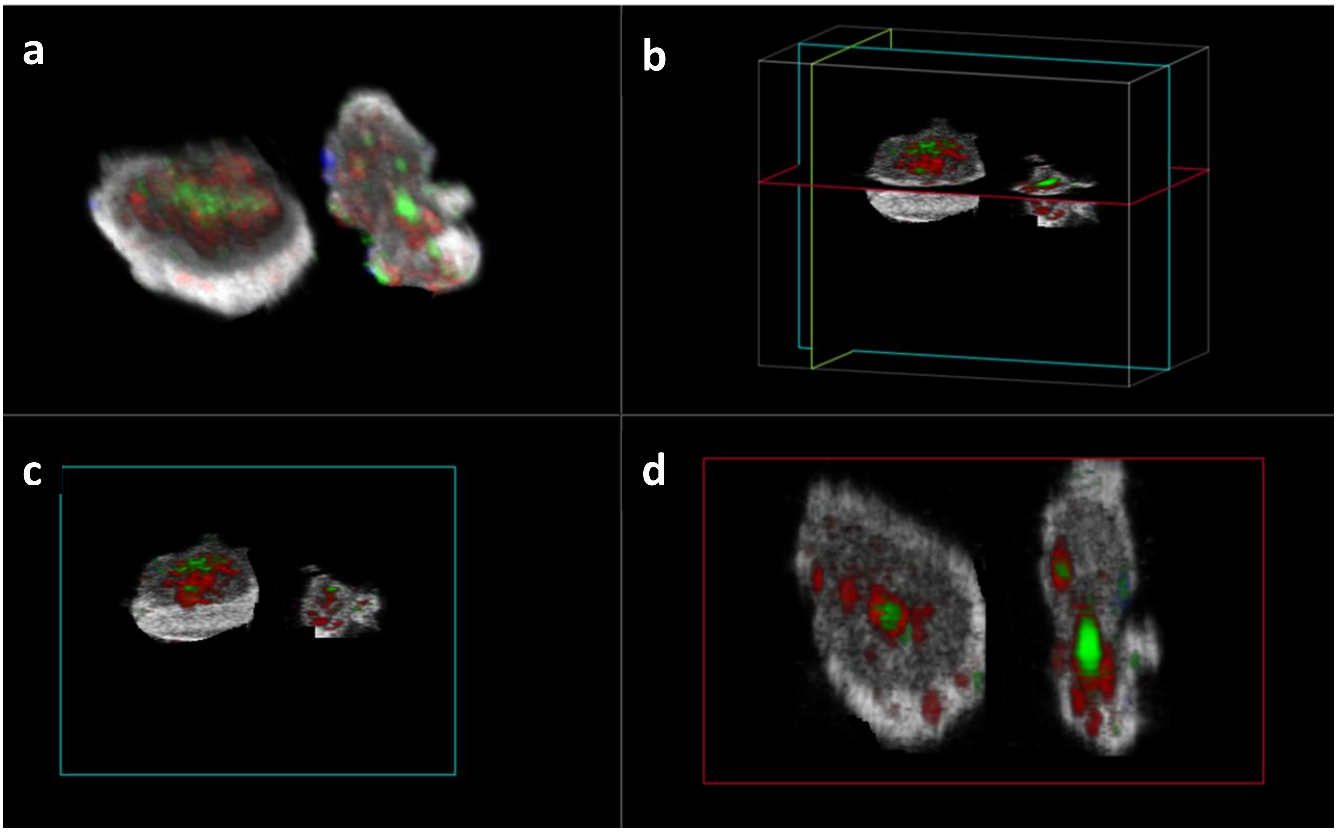

Supplementary Figure 7

3D PAUS volume reconstruction of melanoma samples harvested from a mouse 1 one day after the treatment,acquired with 120 µm motor-step and analyzed in post-processing by spectral unmixing algorithms we reported the:

(a) whole 3D melanoma masses;

(b) 3D texture mapping of three section of interest inside melanoma masses;

(c) maximum signal intensity sagittal slice view of melanoma masses;

(d) maximum signal intensity transverse slice view of melanoma masses; in green color the PA signal from AuNPs-ECFC, in red color the PA signal of oxygenated hemoglobin, in blue color the PA signal of deoxygenated hemoglobin.

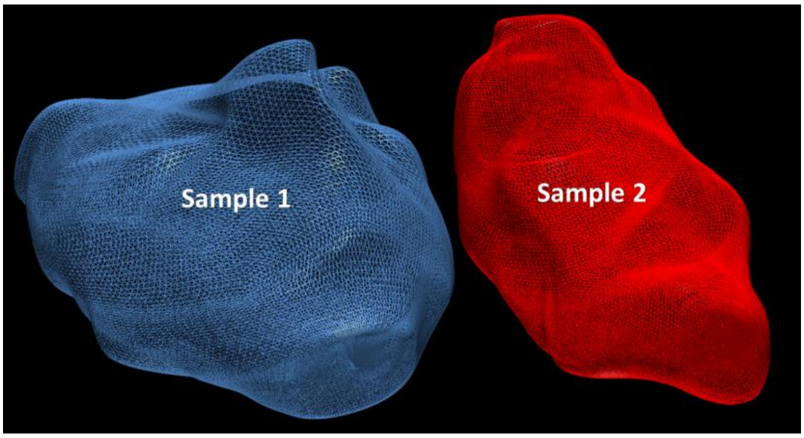

Supplementary Figure 8

Example of 3D volume segmentation for the calculation of melanoma measures,

Summary Table of melanoma measurements

Melanoma masses (time-animal)	%PA Signal$_{\mathrm{AuNPs-ECFC}}$	Volume (mm3)
	**1 Day**	
1day-Sample 1	20	304
1day-Sample 2	16	191
1day-sample 3	28	180
1day-sample 4	31	19
1day-sample 5	11	117
1day-sample 6	25	219
1day-sample 7	17	50
1day-sample 8	17	38
	**1 Week**	
1week-Sample 9	10	434
1week-Sample 10	28	1094

Table SI 2: Calculation of percentage amount of PA signal provides from AuNPs-ECFC per sample on the total amount of PA signal acquired, and measurements of melanoma volumes.

The values of PA Signal were extrapolated after post processing unmixing algorithm on the acquisition, in which the main spectral components (oxy and deoxy hemoglobin and AuNPs-ECFC) were spectrally weighted in the whole 3D volume acquired. The calculation of %PA signal was calculated following the formulation reported below (F1).

$$\%PA\ Signal_{AuNPs-ECFC} = \frac{PA\ Signal_{AuNPs-ECFC}}{PA\ Signal_{total}}\ x\ 100 \quad \text{(F1)}$$

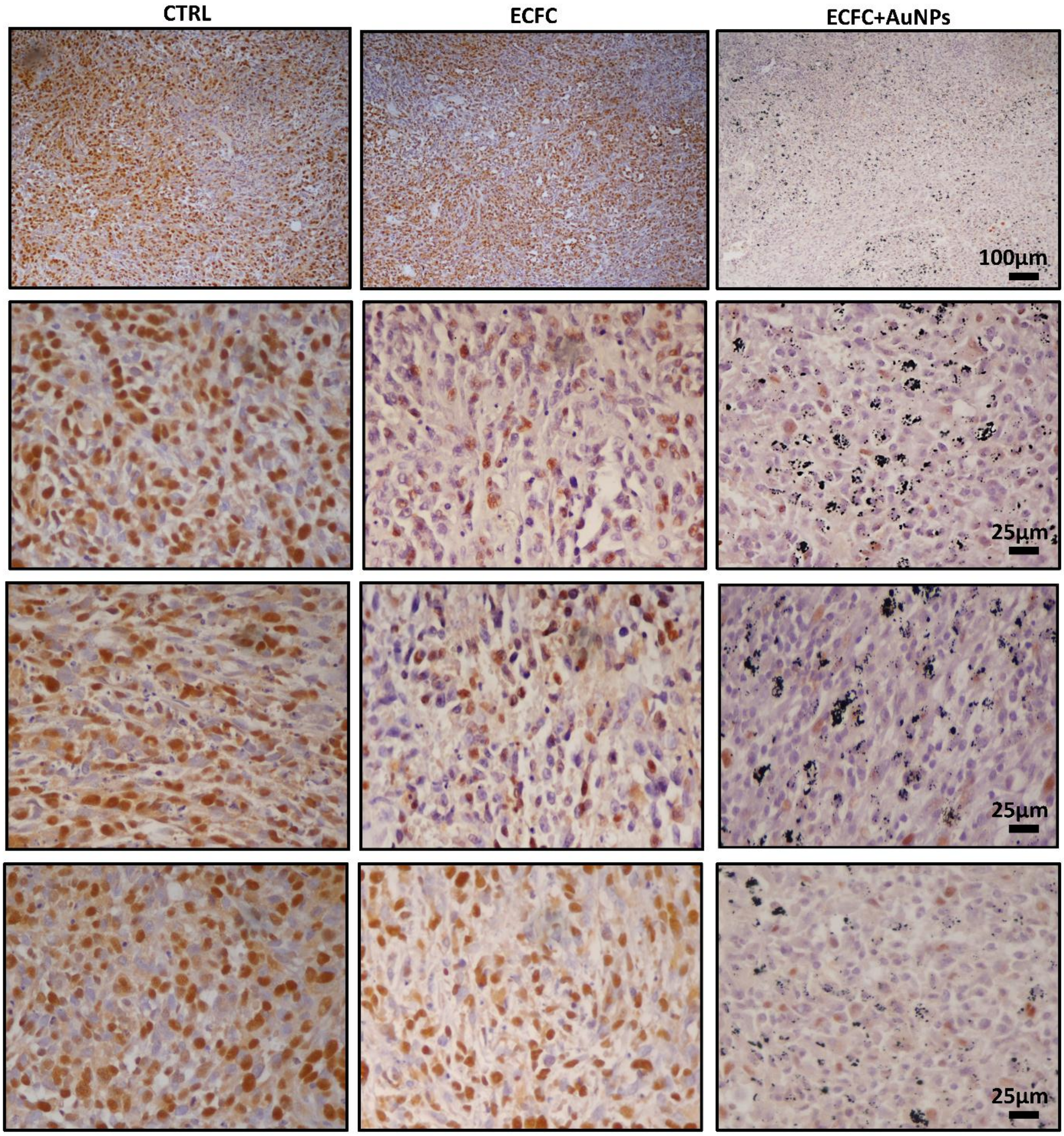

Supplementary Figure 9

Rapresentitative images of PCNA staining on the four tumor masses at different magnifications. First row 10x;second, third and fourth rows 40x.
